# Genome-wide analysis of the genetic regulation of gene expression in human neutrophils

**DOI:** 10.1038/ncomms8971

**Published:** 2015-08-10

**Authors:** Anand Kumar Andiappan, Rossella Melchiotti, Tuang Yeow Poh, Michelle Nah, Kia Joo Puan, Elena Vigano, Doreen Haase, Nurhashikin Yusof, Boris San Luis, Josephine Lum, Dilip Kumar, Shihui Foo, Li Zhuang, Anusha Vasudev, Astrid Irwanto, Bernett Lee, Alessandra Nardin, Hong Liu, Furen Zhang, John Connolly, Jianjun Liu, Alessandra Mortellaro, De Yun Wang, Michael Poidinger, Anis Larbi, Francesca Zolezzi, Olaf Rotzschke

**Affiliations:** 1 Singapore Immunology Network (SIgN), Agency for Science Technology and Research (A*STAR), #04-06, 8A Biomedical Grove, Singapore, Singapore; 2 Department of Human Genetics, Genome institute of Singapore (GIS), Singapore, Singapore; 3 Shandong Provincial Hospital for Skin Diseases, Shandong University, Jinan, Shandong, China; 4 Shandong Provincial Institute of Dermatology and Venereology, Shandong Academy of Medical Sciences, Jinan, Shandong, China; 5 School of Medicine, Shandong University, Shandong Provincial Medical Center for Dermatovenereology, Jinan, Shandong, China; 6 Shandong Provincial Institute of Dermatology and Venereology, Provincial Academy of Medical Science, Jinan, Shandong, China; 7 School of Life Sciences, Anhui Medical University, Hefei, Anhui, China; 8 Department of Otolaryngology, National University of Singapore, Singapore

## Abstract

Neutrophils are an abundant immune cell type involved in both antimicrobial defence and autoimmunity. The regulation of their gene expression, however, is still largely unknown. Here we report an eQTL study on isolated neutrophils from 114 healthy individuals of Chinese ethnicity, identifying 21,210 eQTLs on 832 unique genes. Unsupervised clustering analysis of these eQTLs confirms their role in inflammatory responses and immunological diseases but also indicates strong involvement in dermatological pathologies. One of the strongest eQTL identified (rs2058660) is also the tagSNP of a linkage block reported to affect leprosy and Crohn's disease in opposite directions. In a functional study, we can link the C allele with low expression of the β-chain of IL18-receptor (IL18RAP). In neutrophils, this results in a reduced responsiveness to IL-18, detected both on the RNA and protein level. Thus, the polymorphic regulation of human neutrophils can impact beneficial as well as pathological inflammatory responses.

## Abstract (web summary)

Neutrophils are abundant immune cells important for antimicrobial defence and in autoimmunity. Here, by mapping expression quantitative trait loci (eQTL) in neutrophils of Chinese ethnicity from Singapore, Andiappan *et al.* provide a resource for understanding immune-related trait associated genetic variants.

## Graphical abstract

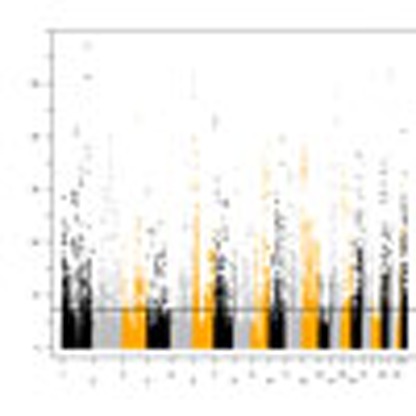

Neutrophils are key players in the innate immune system and crucial for front-line defence against pathogens. They are the first leukocyte subset to migrate to the site of infection, where they recruit other immune cells and activate both the innate and adaptive branches of the immune system^1^,^2^. Neutrophils have several features that equip them to contain bacterial and fungal pathogens including high levels of phagocytosis, the ability to form neutrophil extracellular traps and the capacity to secrete cell-toxic and proinflammatory compounds^3^,^4^. However, these functions must be tightly regulated to avoid excessive destruction of host tissues or the induction of self-reactive immune cells.

On the genetic level this could be achieved by regulatory polymorphisms. genome-wide association studies (GWAS) have found that the majority of allelic variants linked with disease states fall in non-coding regions of the genome^5^,^6^ suggesting that complex pathologies are primarily influenced by genetic control of gene expression. Such non-coding genomic regions with the capacity to affect specific mRNA expression levels are termed ‘expression quantitative trait loci' (eQTLs), and their characterization may thus enable us both to understand disease susceptibility within human populations and to identify important molecular drivers of pathology. Recent reports on eQTLs in isolated immune subsets have yielded strong results providing insights into the genetic basis of disease susceptibility^7^,^8^,^9^,^10^.

In this study we investigated the allelic control of gene expression in neutrophils on the genome wide level and analysed their potential impact on cellular function. We isolated neutrophils from 114 blood samples taken from healthy donors of Chinese descent and performed a genome-wide eQTL analysis to characterize the impact of *cis* polymorphisms on neutrophil transcriptional regulation. The genome-wide correlation of genotype data with gene expression profile revealed more than 800 genes whose expression was affected by single nucleotide polymorphisms (SNPs) within eQTLs in neutrophils.

## Results

**Figure 1:**
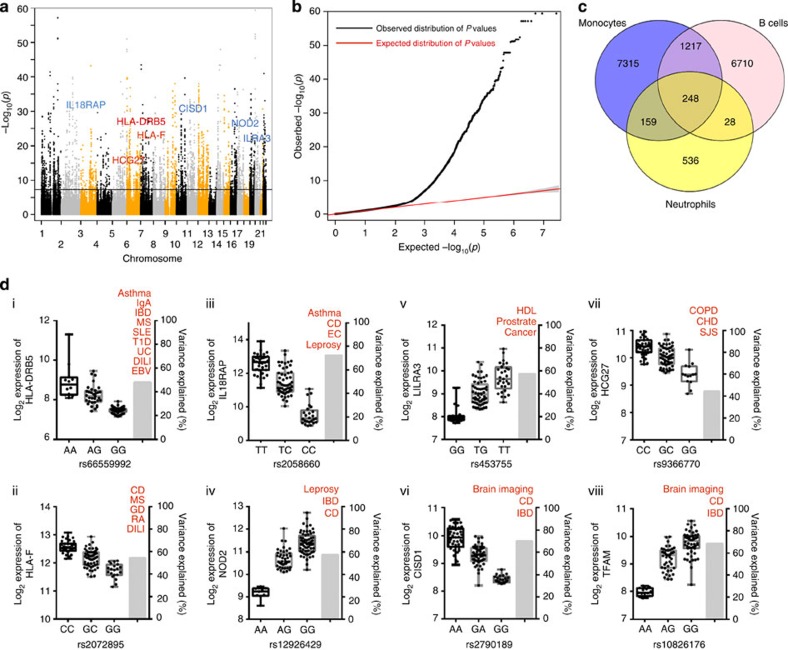
Summary of eQTL identified in neutrophils. (**a**) Manhattan plot of genome-wide distribution, and (**b**) Quantile-Quantile (Q-Q) plot, of the negative logarithm of the association *P* values for eQTLs in neutrophils. Candidates in blue on the panel (**a**) such as IL18RAP, HLA-DRB5, CISD1 and LILRA3 were neutrophil specific, while HCG27, TFAM, HLA-F and NOD2 are shared across other immune cells and displayed in red(**c**) Extent of sharing of eQTLs in neutrophils, B cells and monocytes, using comparative data from (Fairfax *et al.*^7^). (**d**) Selected neutrophil eQTL candidates associated with SNP markers that have been linked with the noted disease phenotypes. Log_2_ expression, error bars for median log_2_ expression and standard deviation are shown (left *y* axis) by SNP genotype (*x* axis) for each of eight genes significant as neutrophil eQTLs together with variance explained (right *y* axis). Crohn's disease (CD), immunoglobulin A (IgA), inflammatory bowel disease (IBD), multiple sclerosis (MS), systemic lupus erythematosus (SLE), type 1 diabetes (T1D), ulcerative colitis (UC), drug-induced liver injury (DILI), epstein-barr virus (EBV) status, total cholesterol measured as HDL, coronary heart disease (CHD), chronic obstructive pulmonary disease (COPD), Stevens–Johnson syndrome (SJS), rheumatoid arthritis (RA), graves disease (GD), eosinophil counts (EC).

By analysing neutrophils from a total of 114 individuals, we identified 21,210 eQTLs that significantly affected 971 distinct probes within 832 unique genes at a permutation significance threshold of *P*<0.001. The Manhattan plot in Fig. 1a shows the negative logarithm of the association *P* value for each eQTL in neutrophils from each donor, across all chromosomes, while the Q-Q plot (Fig. 1b) indicates significant deviations of observed *P* values (linear regression analysis, see Methods for more information) from those expected. A complete list is provided in the (Supplementary Data 1).

### Neutrophil eQTLs shared with other immune subsets

To estimate the extent to which the neutrophil eQTLs were common to other immune cell subsets we compared our data with a published data set containing the cis eQTLs of B cells and monocytes isolated from 288 Caucasian individuals^7^. This data set was particularly interesting because neutrophils and monocytes share a myeloid origin, while B lymphocyte differentiation diverges from myeloid cells at the hematopoietic stem cell level. The definition of eQTL by Fairfax *et al.*, was different from our definition of eQTL as they didn't use the additional permutation *P* value cutoff and thus the difference in the number of eQTLs. When using a linkage disequilibrium threshold of *r*^2^=0.8, only 248 of the 971 eQTLs (25.54%) are found in all three immune subsets (Fig. 1c). This number was significantly larger than expected by chance with a *P* value of 1.5 × 10^−105^ computed using a hyper geometric distribution. The neutrophils shared a greater number of eQTLs with monocytes (159 or 16.37%) as expected, than with the less-related B cells (28 or 2.88%). However, majority of the detected eQTLs (536 or 55.20%), were only differentially expressed in neutrophils and not in B cells or monocytes. The information on the overlapping probes and their corresponding SNPs are provided in (Supplementary Data 3 and 4.

### *In silico* pathway analysis of neutrophil eQTLs

To explore the potential functional implications of the eQTLs detected in neutrophils we used QIAGEN's Ingenuity Pathway Analysis (IPA) tool to identify the biological pathways and processes that were most represented in our eQTL data set (Table 1). In line with the crucial role in pathogen defence, neutrophil eQTLs were significantly enriched in genes participating in the induction of the inflammatory response and/or the manifestation of inflammatory diseases Table 1. This enrichment was in comparison with the entire gene set of all human genes as catalogued by IPA. A significant enrichment was also detected for genes associated with dermatological diseases and disorders as estimated from the *P* value of 1.46 × 10^−4^–2.48 × 10^−2^ (69 eQTLs).

While the enrichment of neutrophil-specific eQTLs in genes involved in immune-related pathways was expected, their marked abundance in skin disease-associated genes was somewhat unexpected and intriguing. We therefore further investigated the association of neutrophil eQTLs with dermatological syndromes: IPA revealed that 43 of the neutrophil eQTLs were associated with psoriasis, 35 with dermatitis, 26 with atopic dermatitis and 2 with dermatitis of ear (Table 2). The enrichment of neutrophil eQTLs was particularly marked for both psoriasis and dermatitis, where association *P* values (IPA based enrichment analysis) reached 8.1 × 10^−4^ and 1.5 × 10^−4^, respectively. Taking this analysis further, we interrogated gene expression data comparing individuals with psoriasis with healthy controls (GEO data set GSE13355 for psoriasis^11^); interestingly, 41 of the 43 neutrophil eQTL associated genes linked with psoriasis in our data were also differentially expressed in psoriasis versus healthy skin samples of the two groups (Supplementary Fig. 1). The corresponding *P* values and log_2_ fold change in gene expression data are provided in Supplementary Table 2. Similar results were also obtained using a second cohort (Gene Expression Omnibus data set GSE14905 ref. 12) where 34 probes out of the 43 were significantly differentially expressed in psoriasis after false discovery rate correction ((Supplementary Figure 2 and Supplementary Table 3). The analysis of a small gene expression data set from doctor-diagnosed dermatitis patients (GSE5667 ref. 13) did not reveal any association with our neutrophil eQTLs, but confirmation using a larger cohort would be necessary to draw any firm conclusions.

### Neutrophil eQTLs linked to GWAS defined disease markers

Given the diverse immunological roles of neutrophils we next asked more broadly whether neutrophil eQTLs were associated with any other disease states in the human population. We exploited the complete Catalog of Published Genome-Wide Association Studies (GWAS ( www.genome.gov/gwastudies) that reports 14947 SNPs taken from 2071 publications considering varied pathological conditions. To facilitate maximum retrieval of possible associations and to limit the impact of differences in ethnicity and varying use of tagSNPs in the genotyping platforms, we extended our set of 971 neutrophil eQTLs by including SNPs that were linked by an *r*^2^ value exceeding 0.8. This combined analysis of the GWAS data sets revealed 89 diseases/traits that were associated with one or more genes linked to neutrophil eQTLs (Supplementary Table 1 and Supplementary Data 2). This was confirmed as significant by enrichment analysis using a hyper geometric distribution for the probability of observing the overlap of the genes affected by the eQTLs against the frequencies reported in the GWAS catalogue (Supplementary Table 4). Among the identified conditions, autoimmune diseases were particularly well-represented: Crohn's disease, multiple sclerosis, ulcerative colitis, inflammatory bowel disease (IBD), as well as systemic lupus erythematosus, Grave's disease, type 1 diabetes and vitiligo were all significantly associated with genes linked to the set of neutrophil eQTLs (Table 3).

Several neutrophil eQTLs were associated with GWAS variants that had been linked to more than one disease/trait, suggesting a more widespread functional impact. The contribution to disease manifestation as well as the genotype-effect on gene expression for some of the more prominent neutrophil eQTLs is shown in Fig. 1d. With the exception of the two MHC genes, *HLA-DRB5* and *HLA-F*, all of these eQTLs were expressed in neutrophils but not monocytes or B cells (compare Fig. 1c). The targeted genes are involved in immune-recognition/regulation and mitochondrial function: HLA-DRB5 encodes the β-chain of one of the common MHC class II molecules; HLA-F is a non-classical class I-like molecule that was recently identified as a ligand for killer inhibitory receptors on NK cells^14^; IL18RAP is the α-chain of the IL18-receptor; NOD2 is a pattern recognition receptor for bacterial peptidoglycans; LILRA3 can bind to MHC class I heavy chains and *HCG27* is a long non-coding RNA gene located in the HLA gene locus. CISD1 is a mitochondrial membrane protein, while TFAM represents a mitochondrial transcription factor. The latter had recently been associated with the modulation of innate immune responses by regulating mitochondrial DNA stress^15^.

### Functional characterization of the IL18RAP eQTL rs2058660

**Figure 2:**
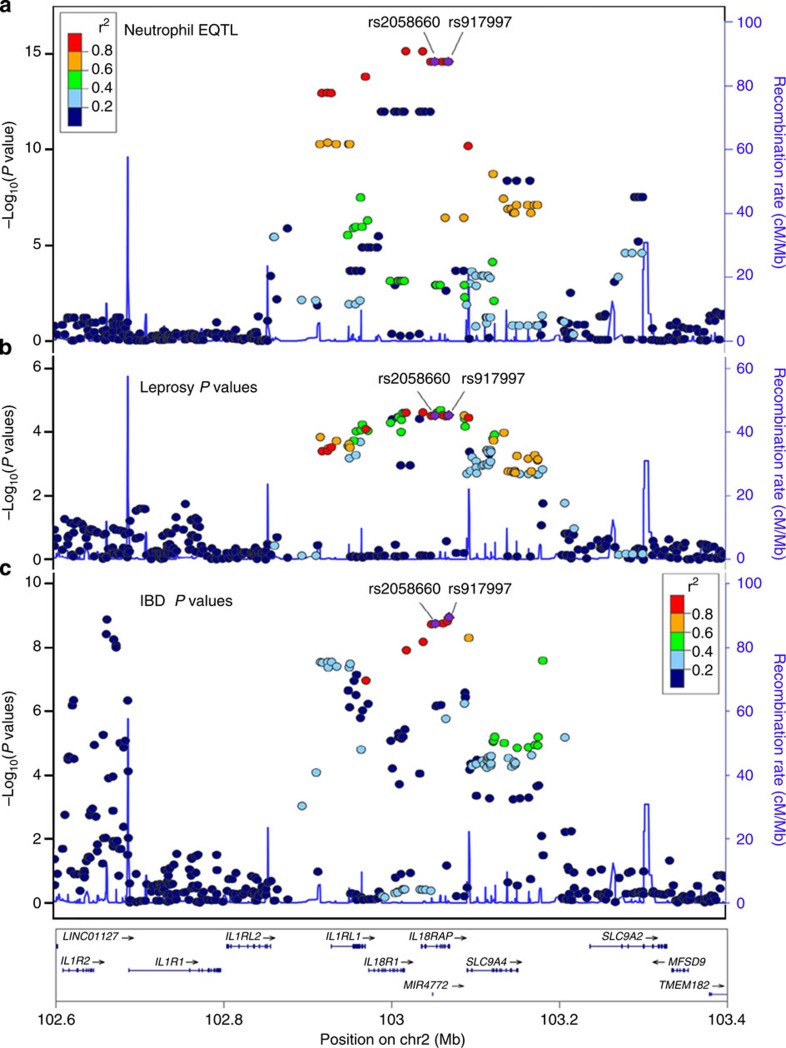
Genetic and functional association of IL18RAP locus SNPs with disease phenotype and possible immune responses. (**a**) A linkage disequilibrium (LD) plot of the IL18RAP region with association *P* values for IL18RAP eQTL in neutrophils observed in this study. (**b**) LD plot of the IL18RAP region with association *P* values for leprosy as observed from Liu *et al* (AJHG, 2012). The disease-associated SNP rs2058660 was replicated at genome-wide significance in the replication cohort with a *P* value of 1.59 × 10^−23^ (**c**) LD plot of the IL18RAP region with association *P* values for IBD as observed from Jostins *et al.*, Nature 2012.

A common feature of all the examples in Fig. 1d is that the eQTL has a strong impact on the respective gene: each account for 40–80% of the observed expression variance of its target gene, implying a major contribution to the disease susceptibility associated with the gene. A particularly interesting example is rs2058660, which accounts for 70% of the variance in *IL18RAP* expression. IL18RAP's ligand, IL18, belongs to the IL-1 cytokine super-family and has a key role in the initiation of proinflammatory immune responses. Accordingly, *IL18RAP* has been identified as a GWAS candidate for asthma and other immune-related diseases including Crohn's disease^16^ and leprosy^17^. A comparison of the negative logarithm association *P* values for SNPs in the gene locus shows that a strong GWAS disease association coincides with a strong effect on neutrophil *IL18RAP* expression (Fig. 2). SNPs exhibiting a low association *P* value in our expression analysis (Fig. 2a), also tended to exhibit low association *P* values in the leprosy GWAS (Fig. 2b) as well as the IBD-GWAS (Fig. 2c) data sets used. This applies particularly for the two SNPs linked to *IL18RAP* expression, rs2058660 and rs917997, which were identified as top candidates based on *P* values in the respective GWAS-studies. For the leprosy GWAS the *P* values displayed in Fig. 2b relate only to the discovery GWAS data and hence the *P* values do not reach genome-wide significance. However, rs2058660 was replicated at genome-wide significance^17^ along with the replication cohort with a *P* value of 1.59 × 10^−23^.

**Figure 3:**
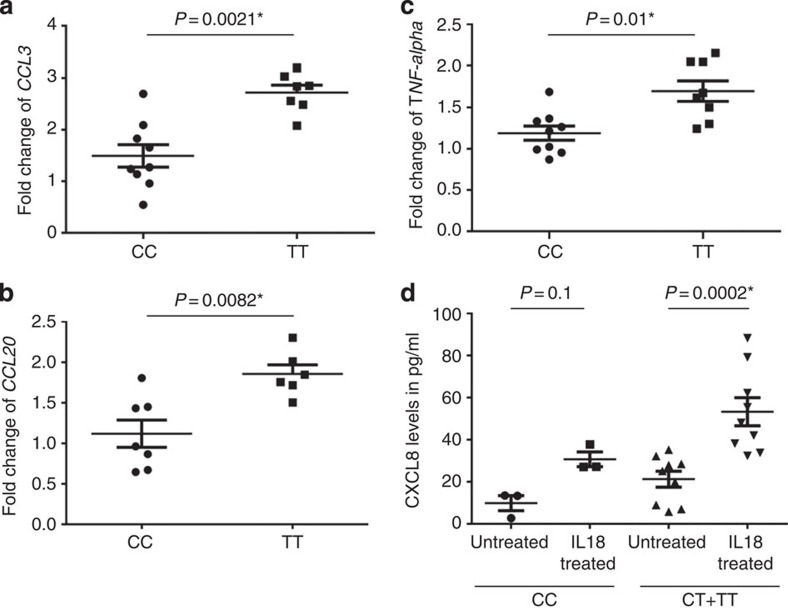
Functional impact of the IL18RAP SNP rs2058660 genotypes. A total of 19 donors were used for this experiment with *n*=7 for TT and *n*=9 for CC for the real time experiments. The gene expression of (**a**) *CCL3* (**b**) *CCL20* (**c**) *TNF-α* following treatment of neutrophils with IL-18 for 14 h, are displayed as a fold increase relative to unstimulated samples, with median and error bars for fold change of the respective gene expression values. *P* values shown are using a non-parametric Mann–Whitney test (**d**) CXCL8 levels measured by ELISA in the supernatant of the neutrophils cultured with IL-18 for 14 h. A total of 13 individuals was used for this experiment with *n*=4 for CC, *n*=6 for CT and *n*=3 for TT. The Kruskal–Wallis involving all groups was significant at *P*=0.001*. *P* values displayed in the figure D are pair wise Mann–Whitney test for each group between treated and untreated.

Therefore, we went on to investigate the functional implications of the allelic state of rs2058660 on IL18-mediated downstream effects in neutrophils. The C allele is associated with low expression of *IL18RAP* (Fig. 1d), and according to the GWAS data, the ‘*TT*' genotype is linked with a higher incidence of leprosy, while ‘*CC*' is the risk genotype for IBD. Considering that an elevated immune response promotes pathogen defence, but also increases the risk of autoimmune reactions, we hypothesize that the risk pattern observed might be explained by a compromised immune-response in low-IL18RAP-expressing T allele individuals. Fortin *et al.*^18^ had also indicated that IL-18 can contribute to generation of several inflammatory cytokines in neutrophils. To test whether this inflammatory cytokine production was dependent on our disease-associated variant, we isolated neutrophils from a total of nine individuals with the ‘*TT*' genotype and eight individuals with ‘*CC*'. We then stimulated the neutrophils overnight with IL-18 before measuring the abundance of proinflammatory gene transcripts (Fig. 3). The real-time PCR for *CCL3*, *CCL20* and *TNF-α* transcripts revealed significantly different expression levels between the two genotypes: in line with lower IL18RAP expression, exposing neutrophils from ‘*CC*' genotype individuals to IL-18 resulted in smaller increases in transcription of the respective cytokines and chemokines. The same trend was evident for IL-8 secretion, as ‘*CC*' individuals exhibited significantly smaller increases in IL-8 release in response to IL-18 than did ‘*CT*' or ‘*TT*' individuals. Thus, the *IL18RAP* eQTL clearly correlates with the induction proinflammatory response genes possibly by varying the amount of the IL18-receptor on the surface of neutrophils.

## Discussion

Recent years have seen a dramatic increase in the number of reported genetic variations and SNPs have been implicated in susceptibility to a wide variety of diseases. The majority of the SNPs fall into non-coding regions of the genome, implying that the main function of these variations is the control of gene expression as opposed to variations in the gene product itself. Interestingly, the majority of eQTLs also appear to be cell type-specific^7^,^19^. Albeit the latter may be partly be affected by different thresholds and is possibly also compounded by the ‘winners curse'^20^ more than half of the neutrophil eQTLs reported here were not present in either monocytes or B cells, and had not been reported before. Some of the neutrophil-specific eQTL genes discovered here have already been validated in a meta-analysis of full blood-eQTL in an computational bioinformatics analysis^21^, which detected 237 of our neutrophil eQTLs. Another whole blood transcriptome study^22^ had identified 10,914 cis eQTLs which overlapped with 699 of the 832 genes identified in our study based on purified neutrophils. While on one hand it confirms our data, it also revealed 133 new genes (16%) that were uniquely defined in our data set. Moreover, as the full blood sample contains many other leukocytes, such monocytes and lymphocytes, a fair number of the eQTLs could initially not be related to neutrophils. Hence, a definite assignment to neutrophils is only possible when purified cells are used. Interestingly a mouse eQTL study by Mostafavi *et al*^10^ detected 84 (10.3%) of our 818 neutrophil eQTLs which could be mapped between human and mouse. This further validates our results on the relevance of these neutrophil eQTLs in other species too. More information on the analysis of comparison of the various data sets has been provided in the methods section and in the online data set file 5.

Since the overlap of eQTLs even between related subsets was somewhat limited, the number of known eQTLs can represent only the tip of the iceberg; this applies even more so as many take effect only under certain functional conditions, such as in response to specific environmental stimuli^23^,^24^,^25^,^26^. Considering further the huge variety of different tissues and cellular subtypes forming the human body, a rather large fraction of genes is likely to be regulated by eQTLs and thus the collection of eQTL data for a selected cell subset can be considered highly informative on its function in health and disease.

We conducted an unbiased pathway analysis (IPA) which revealed an enrichment of neutrophil eQTLs in natural inflammatory responses and in inflammatory diseases consistent with the established role of neutrophils in host defence against pathogens and upon tissue damage^27^. Less expected, however, was the enrichment of neutrophil eQTLs in pathways of dermatological diseases. Further analysis of the neutrophil eQTL-targeted genes in two public databases of psoriasis gene expression revealed that a large proportion was significantly differentially expressed between psoriatic cases and controls. Psoriasis is an immune-mediated disorder characterized by epidermal hyper-proliferation^28^, and neutrophils are known to accumulate under the stratum corneum of highly inflamed psoriatic lesions, where they activate T cells and promote the growth of epidermal keratinocytes^29^,^30^. Our eQTL analysis strongly supports a major contribution of the neutrophil immune cell subset to the pathology of psoriasis, and may be informative for further studies on the underlying mechanisms. A recent meta-analysis of psoriasis-related transcriptome and GWAS data sets also supports this claim. A significant fraction of the psoriasis GWAS candidates showed maximal expression in neutrophils thereby emphasizing its role in immunity^31^.

Besides pathway analysis, the correlation of neutrophil eQTLs with existing GWAS data is another approach to determine function and disease-implication of the subset in an unbiased way. Within a GWAS catalogue covering more than 2,000 syndromes and conditions^32^, significant overlap was detected between neutrophil eQTLs and genes associated with 89 phenotypes. A significant enrichment was also seen in GWAS for autoimmune conditions such as Crohn's disease, ulcerative colitis, systemic lupus erythematosus, Graves's disease, type 1 diabetes and vitiligo. In this study, we took *IL18RAP* as a candidate gene and showed that the differential expression levels that were associated with specific genotypes also had functional consequences in terms of modulating neutrophils' ability to produce the inflammatory cytokine profiles crucial for their immune functions^18^. Context-specific effects of the *IL18RAP* genotype on response to various stimuli have also been recently reported for monocytes and myeloid cells^23^,^33^, adding an additional layer of complexity to our understanding of the regulatory processes important for overall functioning of the immune system. Mostafavi *et al.*^10^ demonstrated that some cis QTL are even shared between humans and mice. This implies that allelic regulation of key genes is crucial for the population to maintain evolutionary flexibility to effectively respond to selection pressures. Our observations may therefore help understand how variations in gene expression associate with disease risk for individuals based both on their haplotype and its effect on specific immune cell subsets.

In summary, using a genome-wide cis eQTL analysis we identified numerous genes whose expression was affected by the presence of polymorphic loci in neutrophils. This data set constitutes a valuable resource both for linking disease-associated genetic variants with function, as demonstrated by the strong overlap between eQTLs and GWAS signals, and for identifying diseases where a dysregulation of key neutrophil genes might play a role. Recent literature already expanded the role of neutrophils from simple suicidal killers to sophisticated players in host defense^34^,^35^,^36^, and our study now adds another layer of understanding of their functional complexity as well as highlighting their involvement in dermal diseases. As they are part of an integrated network of immune cells, eQTLs in neutrophils may have important implications for immune function and barrier protection and could contribute to inter-individual differences in disease susceptibility and pathogenesis.

## Methods

### Samples

This study was performed with the approval of the Institutional Review Board (IRB Reference–NUS07-023 and NUS10-445) of the National University of Singapore and is in compliance with the Helsinki declaration. Blood samples for DNA analysis were collected from a cohort of 114 individuals of Chinese ethnicity, collected from random recruitment drives from January to August 2011 from the National University of Singapore with written informed consent. The demographics of the cohort reflects a median age of 21 (Range 18–29) with more 85% of the participants between 18 and 22 years of age and 42% females. All volunteers were free of serious medical conditions as determined by a questionnaire on self-reported medical history for conditions, such as autoimmune and infectious disease. For this study, blood samples were taken from 114 donors of Chinese ethnicity and used for genotype and gene-expression analysis. Genomic DNA was extracted from whole blood samples and SNP genotyping carried out using the Illumina Omni5Quad system. Total RNA was extracted from the purified neutrophils.

### Neutrophil isolation and storage

Following collection into a BD EDTA Vacutainer, 5 ml of blood was pipetted and diluted with 5 ml of sterile phosphate buffer saline (PBS) and underlayed with 5 ml of Ficoll Paque PLUS in a 15-ml Falcon tube. It was centrifuged at 1,800*g* at room temperature for 20 min. Peripheral blood mononuclear cells were aspirated into a 15-ml Falcon tube before addition of 6 ml of 1 × BD Pharm Lyse to remove red blood cells. After incubation for 15 min at room temperature the lysis solution was diluted to 15 ml with RPMI+10% fetal bovine serum (FBS) and tubes centrifuged at 300*g*, at 4 °C for 5 min. The cell pellet was resuspended into 10 ml of RPMI+10% FBS and centrifuged at 300*g*, at 4°C for 5 min. Cells were finally resuspended in 500 μl of RPMI+10% FBS and a 10 μl sample taken for counting using a MACSQuant. Cells were then adjusted to the appropriate concentration for use in experiments as per the relevant methods section. Cell concentration was adjusted to 5 × 10^6^ cells per ml with RPMI+10% FBS. The samples were aliquoted into sterile 96-well U-bottom plates and the remaining cell suspensions were centrifuged at 300*g*, 4 °C, 5 min. The supernatant was aspirated up to ∼100 μl mark and 300 μl of Trizol LS reagent was added before storage at −80 °C. This preparation of neutrophil samples possibly has a contamination of eosinophils of ∼1–4% and 1–2% of lymphocytes.

### RNA extraction from neutrophils

Total RNA was extracted using the double extraction protocol in batches of 24 samples: acid guanidinium thiocyanate-phenol-chloroform extraction (Trizol Invitrogen) followed by mirVana miRNA Isolation Kit. Agilent Bioanalyzer was used to evaluate total RNA integrity and the RNA integrity number was calculated; all RNA samples had a RNA integrity number ≥ 6.5. DNase digestion was not required since the reverse transcription (RT) reaction uses Oligo DT primers that bind specifically to the polyA tail of the RNA.

### Neutrophil stimulation experiments

For the stimulation experiments neutrophils were isolated from blood as described above, within 4 h of its collection. Briefly, whole blood was diluted with an equal volume of PBS and the peripheral blood mononuclear cells and neutrophils were separated using 1,077 g ml^−1^ ficoll-plaque plus gradient density. The top layer of interphase cells were removed, leaving the behind the neutrophils and erythrocytes. The erythrocytes were then lysed in an ammonium chloride-potassium buffer and the remaining neutrophils resuspended in RPMI1640 supplemented with 10% heat-inactivated FBS and 2 mM L-glutamine (1 × 10^6^ cells/ml). Neutrophils were then treated for 14 h in the absence or presence of 100 ng ml^−1^ IL-18 or 100 ng ml^−1^ lipopolysaccharide. For the subsequent analysis by real-time PCR or enzyme-linked immunosorbent assay, RNA was isolated using Trizol. Real-time PCR was performed using BIO RAD RT-PCR machine. Real-time PCR data were expressed as delta CT, the difference in Ct between the gene of interest (goi) and the endogenous control (end ctl) for a given sample: dCt=Ct(goi)—Ct(end.ctl). The delta CT is then converted to linear fold change in gene expression using the following formula 2(-dCt). Results are then expressed as fold-change between the IL-18 treated samples against the mocked-treated samples between the genotypes.

### Cell supernatant measurements

Neutrophils isolated and stimulated with IL-18 overnight for 14 h and then cell-free supernatants were collected. We then performed quantitative measurements of CXCL8 (Ebioscience, San Diego, California) and CXCL6 (Biolegend, San Diego, California) levels by enzyme-linked immunosorbent assay according to manufacturer's instructions.

### Genome-wide gene expression

Biotinylated cRNA was prepared according to Illumina Ambion TotalPrep 96 RNA Amplification kit (Part Number 4393543) using 40 ng of RNA in batches of 48 samples.The protocol may be summarized into synthesis of first strand, synthesis of second strand, cDNA purification, *in vitro* transcription, cRNA purification. 750 ng of labelled cRNAs were hybridized on the Illumina HumanHT-12-v4 Expression BeadChip Kit (Catalogue number: BD-103-0204) at 58 °C for 16 h. Samples are re-randomized for hybridization and were carried out in batches of 96 samples. Probe sequences provided by Illumina were mapped to genome build hg18 using the pipeline RUM ( http://cbil.upenn.edu/RUM/). Of the total 47,323 probes found on the gene expression microarray a total of 41,469 probes uniquely mapped by the RUM pipeline to hg18 using the default parameters in the RUM pipeline. A total of 39,596 probes were located on the autosomes and retained for the analysis. We did not remove the probes which had low detection in samples as they did not have a major impact on the conclusions and also could be biologically interesting candidates due to the selective loss of expression in some individuals. However, we have flagged those probes which have expression in <5% samples in the online data set file 1. Quantile normalization without background subtraction was then performed using Genome Studio. Expression values were log_2_ transformed before analysis. Probes containing common SNPs (MAF≥1%) according to the 1000 Genome Pilot Project^37^ for CHBJPT were excluded from the analysis (2,467 probes).Clustering was performed to check for any oblivious batch effects and none were observed. Furthermore, a duplicate was detected as it showed an *r*^2^>0.98 and hence was removed from further analysis. Principal component analysis of the expression data reveals that the first 4 PC accounts for 26% of the variance and PC and gender correction had minimal change on the detected eQTLs.

### DNA extraction

DNA was extracted from the blood samples using Qiagen DNeasy Blood & Tissue Kit (Catalogue number: 69504) according to manufacturer's instructions.

### Genome-wide SNP genotyping and quality control

Illumina Human Omni5Quad that can detect up to 4.3 million SNPs was used for genotyping of SNPs. DNA samples were quantified using PicoGreen assay (Molecular Probes) and 400 ng of DNA were used as starting material. Amplifications and hybridizations were performed following the Infinium HD Assay as per manufacture's instruction. Genome coordinates provided by Illumina were converted to genome build hg18 (for consistency with respect to probe annotation) using the UCSC's tool liftOver^38^. A total of 1,067 SNPs could not be converted and were removed from the analysis. SNPs located on sex chromosomes, random chromosomes and mitochondrial chromosomes were also excluded from the computations. Finally filtering for monomorphic and low call rate SNPs (<95%) a total of 2,031,824 SNPs were retained for genome-wide cis eQTL analysis.

### Statistical analysis

Genome-wide statistical association between SNPs and gene expression in the discovery cohort was evaluated using linear regression as implemented by the Apache Commons Mathematics Library. Genotypes were coded based on allele counts (0, 2 for homozygous genotypes and 1 for heterozygous genotypes). *P* values reported as zero were recomputed using the python function linregress from the library scipy to achieve a better precision. For each probe only SNPs located ±250 kbp from the probe midpoint were tested for association. To control the false discovery rate significance was determined by 10,000 phenotype permutations (label swapping) as described in Stranger *et al.*^39^, (originally described by Churchill *et al*^40^) as against just raw *P* values. A <SNP, probe> pair was considered significant if its nominal *P* value was lower than the 0.001 tail of the distribution of minimal permuted *P* values across all SNPs tested for a particular probe. Each probe was analysed independently. Samples with missing genotypes for a particular SNP were excluded from the analysis of the corresponding SNP-probe pair. Illumina SNP IDs coded as kgp were mapped to the corresponding rs IDs by chromosomal position. Statistical association between genotype and gene expression (or protein expression) in the validation cohort was computed using a Kruskal–Wallis one-way analysis of variance (three genotypes) or Mann–Whitney (two genotypes). Multiple comparisons were performed using Dunn's multiple comparisons tests (Graphpad Prism 6).

### Ingenuity pathway analysis

To understand the diseases and disorders associated, we performed network analyses of the entire set of neutrophil eQTL using the IPA tool, (Ingenuity Systems, www.ingenuity.com). Enrichment of the 971 unique probes associated with the 21,210 eQTLs with known pathways, processes and diseases was thus performed using IPA. The standard enrichment analysis in IPA was performed with the entire human genome as the background. Enriched pathways, processes and diseases were then extracted from IPA and reported.

For the psoriasis data sets, gene expression data for the 43 genes identified from the previous analysis was included and visualized as networks. The 43 genes associated with psoriasis as detected by IPA was visualized as networks with network connectivity constructed using manually curated data from IPA and gene expression fold changes between normal and disease used to colour the nodes (red for over-expression and green for decreased expression).

### Comparison with other data sets

Supplementary data from Westra *et al*, 2014 (ref. 21), Mostafavi *et al*, 2014 (ref. 10) and Battle *et al*, 2013 (ref. 21) were retrieved electronically and the eQTLs genes extracted using Biovia Pipeline Pilot (720, 1,222 and 10,914 extracted unique gene symbols). The extract gene names were then matched to the neutrophil eQTLs via their gene names to determine the overlaps between the various studies. In the case of the mouse data from Mostafavi *et al*, 2014 (ref. 10), the genes were mapped to human gene symbols using the data available in HomoloGene build 68 (1055 mapped human gene symbols). The significance of the overlap was estimated using the hyper geometric distribution in R version 2.15.2.

## Additional information

**Accession Codes**: All microarray data are deposited into Gene Expression Omnibus at the National Center for Biotechnology Information under the accession number GSE70044.

**How to cite this article**: Andiappan, A. K. *et al.* Genome-wide analysis of the genetic regulation of gene expression in human neutrophils. *Nat. Commun.* 6:7971 doi: 10.1038/ncomms8971 (2015).

## Supplementary Material

Supplementary Information Supplementary Figures 1-2 and Supplementary Tables 1-4

Supplementary Data 1 Complete list of neutrophil eQTLs identified in this study with statistics

Supplementary Data 2 Complete list of neutrophil eQTLs which had an association with a SNP to one of 89 traits/diseases identified using the GWAS catalogue.

Supplementary Data 3 Complete list of 407 significant probes in neutrophils (current study) which are validated in fairfax monocyte dataset using rsquared=0.8

Supplementary Data 4 Complete list of 276 significant probes in neutrophils (current study) which are validated in fairfax B cells dataset using rsquared=0.8

Supplementary Data 5 List of genes detected in this study with their overlap in Westra et al, 2014, Mostafavi et al, 2014 and Battle et al, 2013.
In the case of Mostafavi et al, 2014, the mouse gene symbols were mapped to human gene symbols using data from HomoloGene build 68. Comparisons of the overlap between the other two studies with the current data show significant overlaps (P=4.444e-151 for Westra et al, 2014, P=1.287E-08 for Mostafavi et al, 2014 and P=8.159E-91 for Battle et al, 2013). For Mostafavi et al, 2014, the cell type for which the eQTL was detected in is provided.

## Figures and Tables

**Table 1:** Diseases or disorders enriched in the significant neutrophil eQTL data set as identified by ingenuity pathway analysis (IPA).

**Disease or disorder**	***P*** **value**	**Number of eQTL**
Inflammatory response	2.39 × 10^−6^–3.50 × 10^−2^	130
Inflammatory disease	1.46 × 10^−4^–3.23 × 10^−2^	94
Dermatological diseases and conditions	1.46 × 10^−4^–2.48 × 10^−2^	69

**Table 2:** Genes reported by IPA for the category dermatological diseases and conditions.

**Disease**	***P*** **value**	**Neutrophil eQTL genes**	**Number of eQTL**
Psoriasis	8.05 × 10^−4^	ACPP,ACTA2,ANXA1,APOBEC3A,ARG1,ATXN3,C3AR1,CLEC7A,CSTB,CTSB,CTSK,CTSS,DBN1,EIF5,EIF5A,FAM26F,GM2A,GNA15,GSN,HAL,HLA-G,HMOX1,IL16,KDELR2,KRT10,KYNU,LILRB1,MBP,PTPRC,RGS1,RNASE3,S100A12,S100P,SEC23B,SIGIRR,TEK,THBD,TYMP,UBE2I,VNN1,VWF,YWHAB,ZNF91	43
Dermatitis	1.46 × 10^−4^	ACADVL,ANXA1,ANXA5,C3AR1,CAPZB,CCR3,CPNE1,CSK,CYTIP,F2RL1,FAS,FCER1G,FLOT1,GNB1L,GSN,GSR,HLA-DRB5,HNRNPR,IL16,KRT10,NFKB1,PGLYRP1,POLE4,RABGEF1,RNASE3,RNF138,RPS6KA4,SELL,SIGIRR,SPAG1,SPI1,STAT6,SYK,THBD,UBE2I	35
Atopic Dermatitis	1.78 × 10^−2^	ACADVL,ANXA1,ANXA5,CAPZB,CCR3,CPNE1,F2RL1,FCER1G,FLOT1,GNB1L,GSN,HLA-DRB5,HNRNPR,KRT10,RNF138,SPAG1,SPI1,SYK,UBE2I	19
Dermatitis of ear	2.48 × 10^−2^	CYTIP,STAT6	2

eQTL, expression quantitative trait loci; IPA, ingenuity pathway analysis

**Table 3:** Specific list of genes with neutrophil eQTLs which also had a significant GWAS *P* value for an autoimmune condition.

**Autoimmune/inflammatory condition**	**Neutrophil eQTLs**
Crohn's disease	CISD1*, IL18RAP*, TRPT1*, TUFM*, DNLZ, ERAP2, HLA-F, RNASET2, TFAM
Inflammatory bowel disease	CISD1*, HLA-DRB5*, TFAM, UTS2
Ulcerative colitis	HLA-DRB5*, LOC650557*, PIM3*, NFKB1, MANBA, UTS2
Rheumatoid arthritis	ARAP1*, CENTD2*, HLA-F, LOC253039, LY6G5C, RNASET2
Type 1 diabetes	HLA-DRB5*, HOXA5*, LOC650557*, SULT1A2*, TUFM*
Multiple sclerosis	CPT1B*, ECGF1, HLA-DRB5*, HLA-F, TYMP*, XRCC6BP1
Graves disease	HCG4*, HLA-F, RNASET2
Vitiligo	HCG4*, RNASET2
Systemic lupus Erythematosus	HLA-DRB5*, UBE2L3
Behcet's disease	CCR3*
Ankylosing spondylitis	CAST*

eQTL, expression quantitative trait loci; GWAS, genome-wide association studies.

Combining data from the neutrophil eQTLs identified with the GWAS catalogue variations for disease associations at GWAS threshold we found a significant enrichment for autoimmune and inflammatory disease. Refer to supplementary table 1 and supplementary online file 1 for comprehensive list.

^*^refers to the neutrophil eQTLs not discovered in the B cells or monocytes.
