# Supplementary material for: Genome-wide analysis of the genetic regulation of gene expression in human neutrophils
Source: Nat Commun. 2015 Aug 10;6:7971. doi: 10.1038/ncomms8971 (PMC4918343; doi:10.1038/ncomms8971)
Supplement: Supplementary Information — Supplementary Figures 1-2 and Supplementary Tables 1-4 [file ncomms8971-s1.pdf]

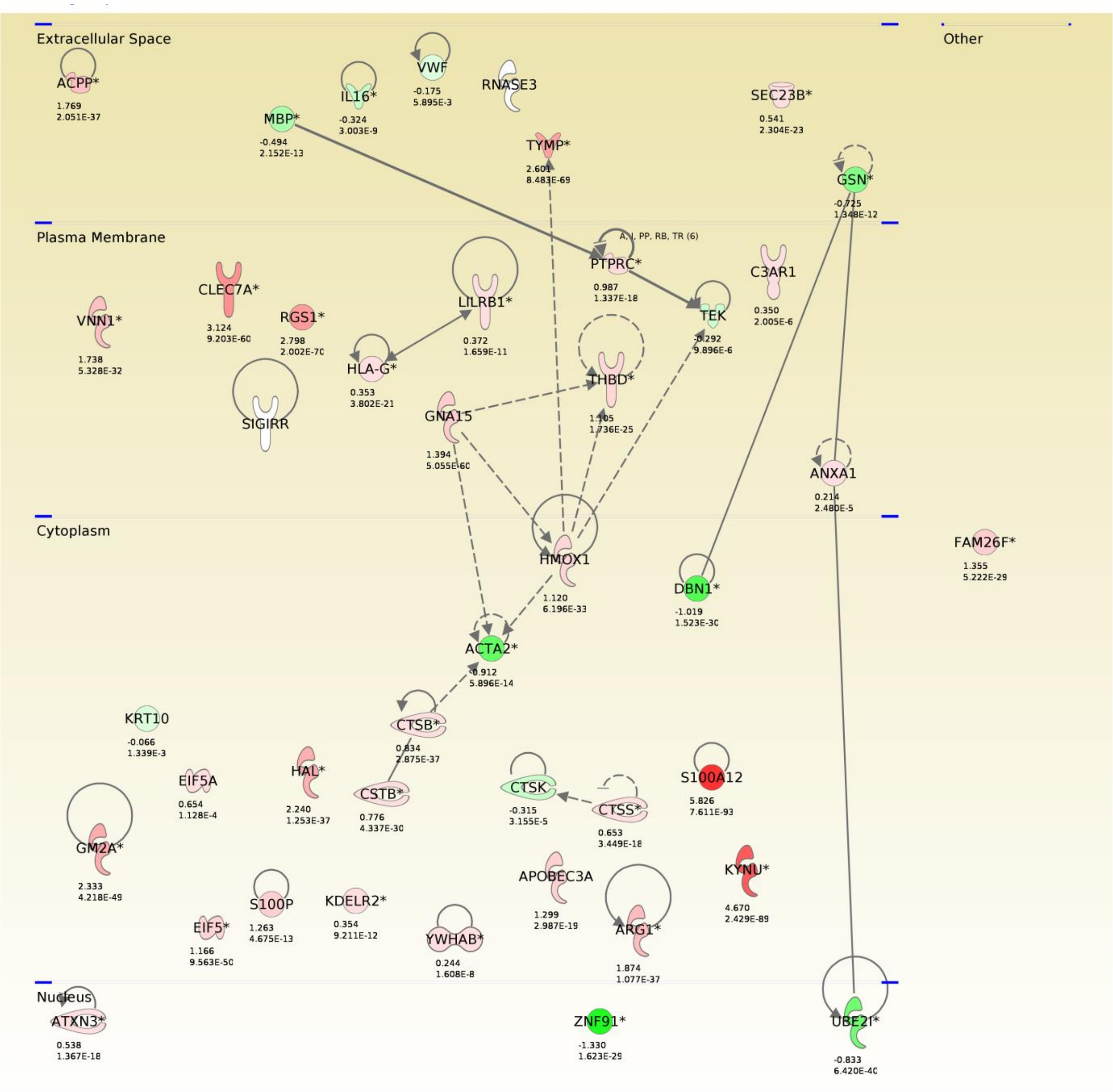

Supplementary figure 1: Network of 43 genes associated with psoriasis identified using IPA. Data was extracted from GEO dataset GSE13355 and significant genes were identified after correcting for multiple testing using false discovery rate (FDR). Connectivity between nodes in the network as well as cellular localization information was obtained from manually curated interaction data from publications provided by Ingenuity Pathway Analysis.

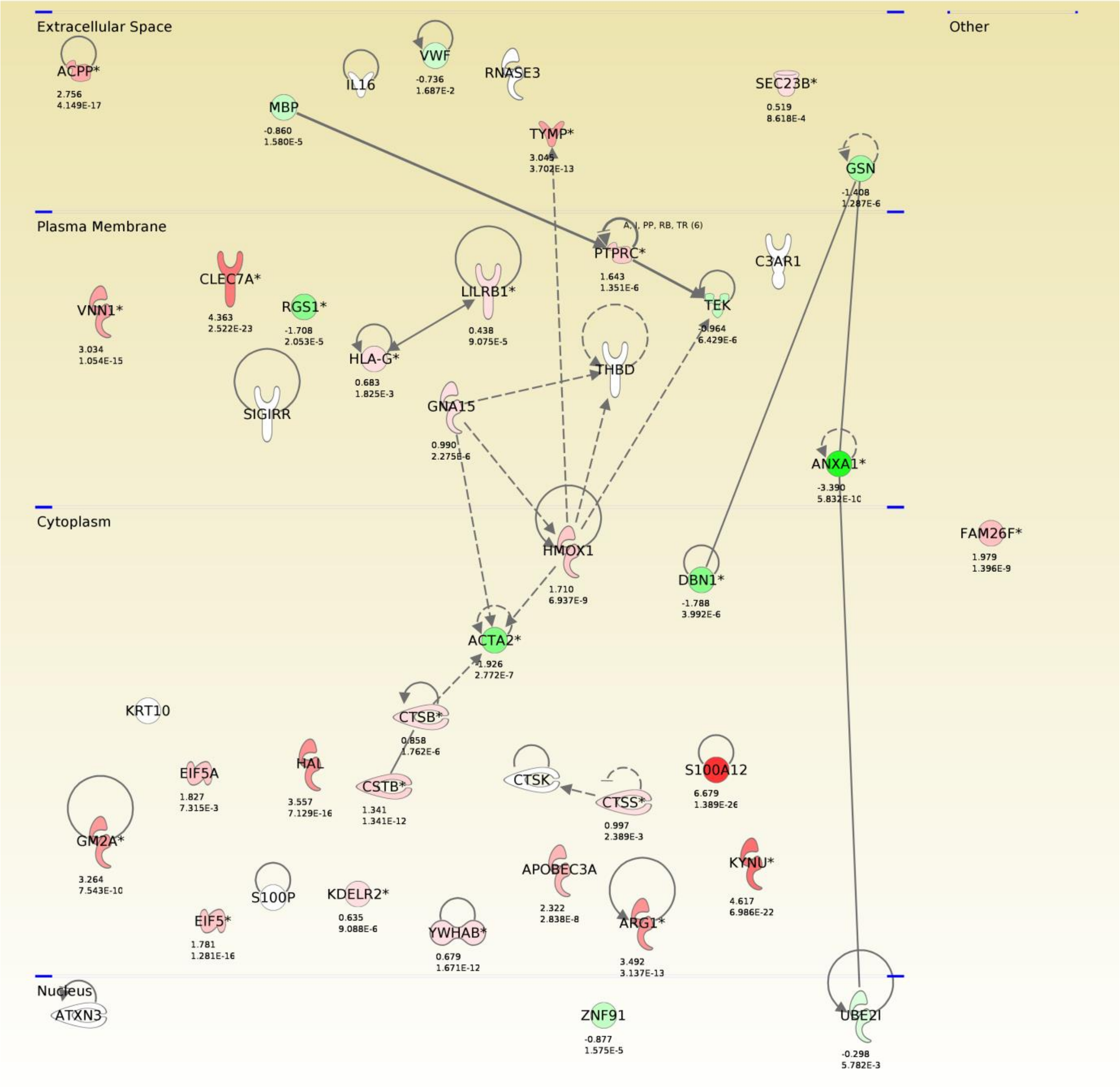

Supplementary figure 2: Network of 43 genes associated with psoriasis identified using IPA. Data was extracted from GEO dataset GSE14905 and significant genes were identified after correcting for multiple testing using false discovery rate (FDR). Connectivity between nodes in the network as well as cellular localization information was obtained from manually curated interaction data from publications provided by Ingenuity Pathway Analysis.

Supplementary Table 1: Overlap between neutrophil eQTLs and GWAS signals group by disease. The 832 HUGO genes identified as significant neutrophil eQTL were then searched for potential association to any GWAS phenotype using the GWAS catalog.

disease	# eQTL genes	eQTL genes	eSNPs	Study	GWAS SNP
Alzheimer's disease	1	MS4A6A	kgp1692058, kgp1982703, kgp6281694, kgp6848150, kgp8004592, kgp8817750, rs12453, rs1441586, rs17602572, rs502581, rs525794, rs540170, rs581133, rs583296, rs610932, rs632185, rs636147, rs662196, rs667897, rs684961, rs688030, rs7926344, rs7935829, rs920573	(Hollingworth et al., 2011)	rs610932
Alzheimer's disease (late onset)	1	MS4A6A	kgp1692058, kgp1982703, kgp6281694, kgp8004592, kgp8817750, rs12453, rs17602572, rs7926344, rs7935829, rs920573	(Naj et al., 2011)	rs4938933
Ankylosing spondylitis	1	CAST	kgp12486558, kgp2211596, kgp3420980, kgp4757267, kgp4972111, kgp5508582, kgp7479499, rs152280, rs2042381, rs26491, rs26510, rs27045, rs27529, rs2911138, rs30187	(Evans et al., 2011; Reveille et al., 2010)	rs27434, rs30187
Asthma	3	HLA-DRB5, IL18RAP, LOC650557	GA023702, GA023720, kgp10004293, kgp10068472, kgp1028867, kgp10300738, kgp10438878, kgp11183228, kgp11545931, kgp11579019, kgp12156514, kgp12402066, kgp1576216, kgp17263711, kgp1774491, kgp2161314, kgp2275154, kgp2281699, kgp2647799, kgp300464, kgp3193328, kgp3705618, kgp3855964, kgp391239, kgp4151029, kgp4501731, kgp4618664, kgp5024284, kgp519135, kgp528534, kgp5334779, kgp543610, kgp6110830,	(Lasky-Su et al., 2012; Moffatt et al., 2010)	rs3771166, rs9272346, rs9273349

			kgp6228000, kgp648083, kgp652537, kgp7104286, kgp7825153, kgp7927966, kgp8004961, kgp8048732, kgp8174959, kgp8234243, kgp8381958, kgp8392648, kgp9414959, kgp9558281, kgp9793551, rs10192036, rs10192157, rs10204837, rs1063355, rs3755276, rs3771166, rs4988955, rs4988956, rs4988957, rs4988958, rs9273349		
Behcet's disease	1	CCR3	kgp2272487, rs2087726, rs7616215	(Kirino et al., 2013)	rs7616215
Beta-2 microglubulin plasma levels	1	BAT5	kgp684920	(Tin et al., 2013)	rs2596466
Blond vs. brown hair color	1	TPCN2	kgp11245254, rs10750836	(Sulem et al., 2008)	rs35264875
Blood pressure	3	C15orf17, CSK, MTHFR	kgp10151543, kgp12435695, kgp1759804, kgp2626044, kgp3256932, kgp3959331, kgp4017153, kgp452259, kgp5856185, kgp7193421, kgp7228049, kgp7588133, kgp9173836, rs11636952, rs12567136, rs13306553, rs13306556, rs13306561, rs1378940, rs1378942, rs1537516, rs17037390, rs17037396, rs17037397, rs17037425, rs17367504, rs17421560, rs2050265, rs2066470, rs2076003, rs2076004, rs2168518, rs2236797, rs2272803, rs2274976, rs3737967, rs3753584, rs7537765, rs7553194	(Wain et al., 2011)	rs1378942, rs17367504
Body mass index	3	MYBPC3, SPI1, TUFM	GA027821, kgp12121075, kgp12178462, kgp12559971, kgp3144475, kgp4095036, kgp5388595, kgp5729725, rs10769258, rs10838698, rs10838708, rs12419692, rs12798346, rs34958982, rs4752845,	(Speliotes et al., 2010; Thorleifsson et al., 2009; Willer et al., 2009)	rs10838738, rs3817334, rs7498665

			rs4752993, rs4752999, rs7124681, rs755553, rs7937331, rs8049439, rs896816		
Brain imaging	2	CISD1, TFAM	kgp11037171, kgp12103589, kgp1941074, kgp21624023, kgp21640455, kgp2165853, kgp21713428, kgp21741288, kgp21766871, kgp22026038, kgp2934343, kgp351673, kgp4000505, kgp5495461, kgp6035958, kgp8040972, kgp8413294, rs1416767, rs16911872, rs16912145, rs2590289, rs2590303, rs7068355, rs7073090, rs7087498, rs965867	(Shen et al., 2010)	rs16912145
Brain structure	2	HRK, TMEM118	rs7132339, rs7133290, rs7294919	Identification of common variants associated with human hippocampal and intracranial volumes.	rs7294919
Breast cancer	1	ECHDC1	kgp3196371, kgp7525672, kgp8519099, kgp8884486, kgp9313574, rs2180341, rs4897207, rs6569476, rs6900074, rs6912006, rs7453272, rs7741162, rs7745944, rs7760748, rs9375503, rs9385424, rs9388568, rs9398840, rs9401953	Genome-wide association study provides evidence for a breast cancer risk locus at 6q22.33.	rs2180341
C4b binding protein levels	1	C4BPA	kgp12121661, kgp4256512, kgp7890677, kgp8310661, rs11120211, rs11120218, rs17020983, rs2808470	C4BPB/C4BPA is a new susceptibility locus for venous thrombosis with unknown protein S-	rs3813948

				independent mechanism: results from genome-wide association and gene expression analyses followed by case-control studies.	
Celiac disease	1	RGS1	kgp1344958, kgp3413464, kgp4299659, rs10754012, rs1323292, rs1323298, rs1323299, rs2760520, rs2760530, rs2816316, rs2984920	Multiple common variants for celiac disease influencing immune gene expression., Newly identified genetic risk variants for celiac disease related to the immune response.	rs2816316
Cholesterol, total	2	, CPNE1	kgp10244699, kgp10523516, kgp12353707, kgp1571375, kgp2263242, kgp7291850, kgp7677608, kgp9271923, kgp9433925, rs11167275, rs12481228, rs12481545, rs13038622, rs17725246, rs2425149, rs6058296, rs6060625, rs6120998, rs7270009	Biological, clinical and population relevance of 95 loci for blood lipids.	rs2072183, rs2277862
Chronic obstructive pulmonary disease-related biomarkers	2	HCG27, PSORS1C3	rs1265093, rs2074488	Genome-wide association analysis of blood biomarkers in chronic obstructive pulmonary	rs1265093, rs2074488

				disease.	
Cleft lip	1	TRAF3IP3	kgp10188595, kgp2058676, kgp6637897, kgp7973574, rs10863790, rs12063989, rs2235371, rs2235377, rs4844496	A genome-wide association study of cleft lip with and without cleft palate identifies risk variants near MAFB and ABCA4.	rs10863790
Coffee consumption	1	CSK	rs2168518	Genome-wide association analysis of coffee drinking suggests association with CYP1A1/CYP1A2 and NRCAM.	rs6495122
Coronary heart disease	1	HCG27	rs3869109	A genome-wide association study for coronary artery disease identifies a novel susceptibility locus in the major histocompatibility complex.	rs3869109
Crohn's disease	10	, CISD1, DNLZ, ERAP2, HLA-F, IL18RAP, RNASET2, TFAM, TRPT1, TUFM	kgp10976490, kgp1110300, kgp11138329, kgp11193800, kgp11343503, kgp11349623, kgp12431362, kgp12482760, kgp12574568, kgp1692957, kgp1753996, kgp1998378,	A genome-wide scan of Ashkenazi Jewish Crohn's disease suggests novel	rs151181, rs1819658, rs2058660, rs2301436, rs2549794, rs4077515, rs415890, rs694739, rs9258260

			kgp2493955, kgp2554877, kgp2819229, kgp2850730, kgp3153393, kgp3245966, kgp3523543, kgp3599615, kgp3829723, kgp3879776, kgp3973174, kgp4006547, kgp4017619, kgp402086, kgp4480527, kgp4792454, kgp4862811, kgp5155271, kgp5388595, kgp5603667, kgp5702444, kgp5870714, kgp6202901, kgp6362205, kgp6486241, kgp6982210, kgp7472568, kgp7473702, kgp7564707, kgp7599113, kgp766516, kgp7850076, kgp8113001, kgp8170133, kgp818352, kgp8636080, kgp8784304, kgp8885175, kgp9014236, kgp9054956, kgp9304010, kgp9400347, kgp9417276, kgp9568439, kgp9670249, kgp9864241, rs1005043, rs1014286, rs10178436, rs10193009, rs10484530, rs1056893, rs10781499, rs10781500, rs10781505, rs10781510, rs10946204, rs11145850, rs11465730, rs11685424, rs11794847, rs1199085, rs1199098, rs1199106, rs12183084, rs12525345, rs12526548, rs12529876, rs12712135, rs12712141, rs13015714, rs1303960, rs1357471, rs1416763, rs1416764, rs1420106, rs1468791, rs1558622, rs1769039, rs1867571, rs1981846, rs2001114, rs2005881, rs2008157, rs2008159, rs204295, rs2058622, rs2058623, rs2058660, rs2110734, rs2110735, rs2149085, rs2153281, rs2161657, rs2181058, rs2251039, rs2254775, rs2272128, rs2282860, rs2287988,	susceptibility loci., Genome-wide association defines more than 30 distinct susceptibility loci for Crohn's disease., Genome-wide meta-analysis increases to 71 the number of confirmed Crohn's disease susceptibility loci.	

			rs2301436, rs239935, rs2486483, rs2486491, rs2548527, rs2549782, rs2549785, rs2549796, rs2590313, rs2653505, rs2790155, rs2790170, rs2790189, rs2790216, rs2790236, rs2790241, rs3812570, rs3812575, rs3829112, rs394522, rs400837, rs4077515, rs4266763, rs429083, rs439553, rs4399750, rs4851609, rs4851610, rs4851613, rs4851619, rs510372, rs6708949, rs6734736, rs6737119, rs6750851, rs6750971, rs6902119, rs6907666, rs6921588, rs7083712, rs741285, rs7559479, rs7568913, rs759382, rs7700332, rs7749278, rs7899961, rs8049439, rs917997, rs9258260, rs932644, rs933243, rs9355610, rs9411205, rs9459836, rs974389		
Dengue shock syndrome	1		rs3132468	Genome-wide association study identifies susceptibility loci for dengue shock syndrome at MICB and PLCE1.	rs3132468
Diastolic blood pressure	3	C15orf17, CSK, LY6G5C	rs11636952, rs1378940, rs1378942, rs2168518, rs805303	Genetic variants in novel pathways influence blood pressure and cardiovascular disease	rs1378942, rs6495122, rs805303

				risk., Genome-wide association study identifies eight loci associated with blood pressure., Genome-wide association study of blood pressure and hypertension.	
Drug-induced liver injury (amoxicillin-clavulanate)	2	HLA-DRB5, HLA-F	kgp10086042, kgp10716828, kgp10791374, kgp10993269, kgp11057669, kgp11097589, kgp11111197, kgp112624, kgp11434010, kgp11488527, kgp11503156, kgp11525948, kgp11670806, kgp11899131, kgp11951896, kgp12220543, kgp12292865, kgp12336002, kgp1497861, kgp1727184, kgp1770756, kgp1830625, kgp1840917, kgp1900289, kgp2124132, kgp2421267, kgp2915226, kgp2944558, kgp3013361, kgp3097457, kgp3302220, kgp3555861, kgp3641442, kgp369526, kgp3844509, kgp398454, kgp4186203, kgp4275121, kgp4330952, kgp4550051, kgp4630866, kgp5219378, kgp5417994, kgp5422503, kgp5522547, kgp5658501, kgp5699920,	Susceptibility to amoxicillin-clavulanate-induced liver injury is influenced by multiple HLA class I and II alleles.	rs2523822, rs9274407

			kgp5786063, kgp5870137, kgp605392, kgp6621603, kgp6754068, kgp6778497, kgp6977253, kgp7045337, kgp7283944, kgp732004, kgp7353290, kgp8041485, kgp8373990, kgp8432767, kgp8438749, kgp8765630, kgp8855468, kgp926303, kgp9265745, kgp9455964, kgp95572, kgp967253, kgp9744894, kgp9751502, kgp9763090, kgp9797931, kgp981634, kgp9983308, kgp9990055, rs2523822, rs9270856, rs9271055, rs9271100, rs9271170, rs9271525		
Endometriosis	2	, CDC42	kgp11139620, kgp1199370, rs2235529	Genome-wide association study link novel loci to endometriosis.	rs10917151, rs4654783, rs2235529
Eosinophil counts	1	IL18RAP	kgp11010155, kgp12099647, kgp12162962, kgp3741642, kgp4269063, kgp6039805, rs12712142, rs13001325, rs1420101, rs17026974, rs1921622, rs2287037	Sequence variants affecting eosinophil numbers associate with asthma and myocardial infarction.	rs1420101
Epstein-Barr virus immune response (EBNA-1)	1	HLA-DRB5	kgp11899131, kgp5522547, kgp5786063, kgp6621603, kgp8432767, kgp8765630, kgp967253	A genome-wide integrative genomic study localizes genetic factors influencing antibodies against Epstein-Barr virus nuclear antigen 1	rs477515

				(EBNA-1).	
Eye color traits	1	C17orf90	kgp10010099, kgp1705580, kgp2532886, kgp501530, kgp6422722, kgp7938492, rs34815730, rs7502869	Digital quantification of human eye color highlights genetic association of three new loci.	rs9894429
Glaucoma (primary open-angle)	1	PIK3C2A	kgp7402249, rs11024102	Genome-wide association analyses identify three new susceptibility loci for primary angle closure glaucoma.	rs11024102
Graves' disease	3	HCG4, HLA-F, RNASET2	kgp11138329, kgp12482760, kgp1753996, kgp2850730, kgp3245966, kgp7564707, kgp766516, kgp818352, kgp9568439, rs10484530, rs10946204, rs12183084, rs204295, rs2282860, rs239935, rs3893464, rs6907666, rs6921588, rs7749278, rs933243, rs9355610, rs9459836	A genome-wide association study identifies two new risk loci for Graves' disease., Identification of independent risk loci for Graves' disease within the MHC in the Japanese population.	rs3893464, rs9355610
HDL cholesterol	8	CDK2AP1, KCTD10, LILRA3, LOC100133875, MYBPC3, PPP1R1B, SPI1,	kgp1008583, kgp10325250, kgp10457578, kgp10574728, kgp11111000, kgp11130257, kgp1114377, kgp11496797, kgp12162709, kgp12495023, kgp1727166,	Biological, clinical and population relevance of 95 loci for blood lipids., Common	rs11869286, rs181362, rs2338104, rs386000, rs4759375, rs7120118

		UBE2L3	kgp2504636, kgp3148231, kgp3401301, kgp3518509, kgp3671882, kgp3719173, kgp4194516, kgp4304107, kgp4496970, kgp457143, kgp483628, kgp4915908, kgp499544, kgp5158011, kgp5425760, kgp5759935, kgp6117999, kgp6674207, kgp6806053, kgp6927231, kgp7170539, kgp7616660, kgp787031, kgp796636, kgp8307710, kgp8533041, kgp8746728, kgp8871690, kgp8893061, kgp9630336, kgp9720238, kgp998131, rs10161126, rs103294, rs1034329, rs10501321, rs1052373, rs10773000, rs10773003, rs10838687, rs10838692, rs10846489, rs10846519, rs10846523, rs10850379, rs10852934, rs11057223, rs11078919, rs11089629, rs11089637, rs1109559, rs1109560, rs11570094, rs11609896, rs12150298, rs12158299, rs12314392, rs131665, rs140489, rs140492, rs140498, rs1449627, rs1476278, rs1565922, rs1568428, rs1615350, rs1727293, rs1727301, rs1727302, rs181360, rs181362, rs1879379, rs2070512, rs2167079, rs2266959, rs2283790, rs2305280, rs2517955, rs2517956, rs2517957, rs28414347, rs28501453, rs28533432, rs28587386, rs28595660, rs28660993, rs2934952, rs2941504, rs326214, rs326217, rs3758673, rs3764352, rs3803001, rs3816725, rs3847502, rs4752979, rs4759377, rs4795388, rs4821112, rs4821116,	variants at 30 loci contribute to polygenic dyslipidemia., Genome-wide association analysis of metabolic traits in a birth cohort from a founder population., Newly identified loci that influence lipid concentrations and risk of coronary artery disease.	

			rs5754217, rs7120118, rs7297649, rs7315453, rs738127, rs7395581, rs7959415, rs7972811, rs798887, rs830085, rs861857, rs878825, rs879606, rs901746, rs903501, rs903502, rs9303274, rs931992, rs9795593, rs9972882		
Height	9	CCBL2, CLIC4, FIG4, N4BP2L2, OCEL1, PFAAP5, PIK3C2A, RFP, SMPD2	GA024053, kgp10073063, kgp10619656, kgp10895873, kgp10985442, kgp11821945, kgp12431176, kgp12521225, kgp2052635, kgp2514780, kgp3130001, kgp3425874, kgp3455195, kgp3552105, kgp3625443, kgp4261283, kgp4308798, kgp4383562, kgp4603388, kgp4905738, kgp513097, kgp5144493, kgp5753668, kgp6377880, kgp6560572, kgp6730652, kgp6880103, kgp7196136, kgp7537520, kgp7984957, kgp8316735, kgp8752111, kgp9579267, kgp9731946, rs10457192, rs10782163, rs10903104, rs10903106, rs11153183, rs11249177, rs11755641, rs11840502, rs12428283, rs12439, rs1316829, rs1324414, rs1330, rs1476387, rs1890449, rs1924606, rs1929937, rs214105, rs2183873, rs2279007, rs2279008, rs2301390, rs2301393, rs3129109, rs3131502, rs3742320, rs3752474, rs3890756, rs3899233, rs4057297, rs4437822, rs4562565, rs4601530, rs4648878, rs4942791, rs4942829, rs4942892, rs499966, rs6604979, rs6667299, rs6911700,	Hundreds of variants clustered in genomic loci and biological pathways affect human height.	rs1046943, rs1330, rs2279008, rs3129109, rs4601530, rs6699417, rs7332115

			rs6921615, rs7318510, rs7318563, rs7328733, rs7332115, rs7519508, rs7526415, rs7528154, rs7535819, rs7548438, rs7555134, rs7748669, rs7754650, rs7755941, rs7985656, rs829811, rs910730, rs9315167, rs9372223, rs9374111, rs9384719, rs9386822, rs9386829, rs9398210, rs9487069, rs9591143, rs9591191, rs9595617, rs9595630, rs9595795, rs9595908, rs9595946, rs9596218		
Hippocampal volume	2	HRK, TMEM118	rs7132339, rs7133290, rs7294919	Common variants at 12q14 and 12q24 are associated with hippocampal volume.	rs7294919
Homocysteine levels	1	MFN2	rs1801133	Novel associations of CPS1, MUT, NOX4, and DPEP1 with plasma homocysteine in a healthy population: a genome-wide evaluation of 13 974 participants in the Women's Genome Health Study.	rs1801133
Hypertension	1	LY6G5C	rs805303	Genetic variants in novel pathways	rs805303

				influence blood pressure and cardiovascular disease risk.	
Hypothyroidism	1	VAV3	kgp11273072, kgp3817360, rs17014208, rs17020055, rs17020088, rs17020104, rs7537605	Novel associations for hypothyroidism include known autoimmune risk loci.	rs4915077
IgA nephropathy	2	CD68, HCG4	rs2523946, rs4968212, rs4968213, rs4968214	A genome-wide association study in Han Chinese identifies multiple susceptibility loci for IgA nephropathy.	rs2523946, rs4227
Immunoglobulin A	1	HLA-DRB5	rs9271366	Association of IFIH1 and other autoimmunity risk alleles with selective IgA deficiency.	rs9271366
Inflammatory bowel disease	4	CISD1, HLA-DRB5, TFAM, UTS2	kgp11193800, kgp11899131, kgp12431362, kgp1692957, kgp2493955, kgp3973174, kgp4017619, kgp4149756, kgp4327462, kgp4792454, kgp5522547, kgp5786063, kgp6621603, kgp7023928, kgp8432767, kgp8765630, kgp9054956, kgp9400347, kgp9670249, kgp967253, kgp9864241, rs10826172, rs11006121, rs1199085,	HLA-Cw*1202-B*5201-DRB1*1502 haplotype increases risk for ulcerative colitis but reduces risk for Crohn's disease., Host-	rs2790216, rs35675666, rs477515, rs9271366

			rs1199098, rs1199106, rs12758337, rs1416763, rs1416764, rs161802, rs17367289, rs1769039, rs1867571, rs1963927, rs2153281, rs225100, rs2251039, rs225106, rs225119, rs225132, rs2254775, rs226242, rs226251, rs2486483, rs2486491, rs2493215, rs2590313, rs2653505, rs2790155, rs2790170, rs2790189, rs2790216, rs2790236, rs2790241, rs397349, rs400736, rs442862, rs7083712, rs7899961, rs9271366	microbe interactions have shaped the genetic architecture of inflammatory bowel disease., Loci on 20q13 and 21q22 are associated with pediatric-onset inflammatory bowel disease.	
Iris color	1	HERC2	kgp1463874, rs1667394, rs8039195, rs916977	Three genome-wide association studies and a linkage analysis identify HERC2 as a human iris color gene.	rs916977
LDL cholesterol	1		kgp1571375, kgp9271923, kgp9433925, rs17725246	Biological, clinical and population relevance of 95 loci for blood lipids.	rs2072183
Leprosy	1	NOD2	kgp2765303, kgp3890691, rs1981760, rs2287195, rs4785223, rs7194886, rs7202124, rs8044354	Genomewide association study of leprosy.	rs9302752
Lipoprotein-associated phospholipase A2 activity and mass	1	MS4A6A	kgp1045455, kgp628708, rs573790, rs600550, rs664034, rs668287, rs670139, rs676309	Genome-wide association study evaluating lipoprotein-associated phospholipase A2 mass	rs600550

				and activity at baseline and after rosuvastatin therapy.	
Liver enzyme levels	1	NRBF2	kgp10632504, kgp10931301, kgp11960072, kgp1877178, kgp3151715, kgp6082641, kgp8777802, rs10761725, rs10761742, rs1553789, rs1935, rs4335432, rs6479889, rs7073746, rs7098614, rs7896783, rs7923609, rs871690	Population-based genome-wide association studies reveal six loci influencing plasma levels of liver enzymes.	rs10761779, rs12355784
Liver enzyme levels (gamma-glutamyl transferase)	3	CCBL2, DDT, THBS3	kgp11624868, kgp12113598, kgp12181828, kgp1501247, kgp2319766, kgp2483354, kgp3205062, kgp3552105, kgp4494561, kgp4683404, kgp7515647, kgp8907458, rs1002286, rs1006771, rs1007888, rs10157801, rs12028043, rs12904, rs2000467, rs2000468, rs2186366, rs2330635, rs4276913, rs4745, rs4822451, rs4822453, rs4822454, rs4822466, rs4971079, rs5760095, rs5760102, rs9297	Genome-wide association study identifies loci influencing concentrations of liver enzymes in plasma.	rs10908458, rs12145922, rs2739330
Lymphoma	1	TAP2	rs2621416	Susceptibility loci associated with specific and shared subtypes of lymphoid malignancies.	rs2621416
Magnesium levels	1	THBS3	kgp10676451, kgp4656286, kgp4859340, kgp8652154, rs11264337, rs11264338, rs11264339, rs2049805, rs2075571, rs2990245,	Genome-wide association studies of serum	rs4072037

			rs3814316, rs4072037, rs4971100, rs914615	magnesium, potassium, and sodium concentrations identify six Loci influencing serum magnesium levels.	
Mean corpuscular hemoglobin	1	DNASE2	kgp12152894, kgp3737207, kgp8672702, rs1010222, rs10451496, rs1060218, rs11085824, rs11085825, rs2965214, rs2974750, rs741702, rs8110787	Multiple loci influence erythrocyte phenotypes in the CHARGE Consortium.	rs11085824
Mean corpuscular volume	1	DNASE2	rs10419408	Multiple loci influence erythrocyte phenotypes in the CHARGE Consortium.	rs7255045
Mean platelet volume	2	C12orf47, NRBF2	kgp10096510, kgp10237880, kgp1156172, kgp11702452, kgp11960072, kgp12301610, kgp1809527, kgp234269, kgp2376114, kgp2869126, kgp4200842, kgp5052123, kgp5458224, kgp5840769, kgp6174673, kgp6423030, kgp6475356, kgp7308998, kgp7865620, kgp8499767, kgp9423517, rs11066054, rs11066077, rs11066089, rs11066118, rs11066119, rs11066128, rs12306814, rs12321677, rs12423041, rs12426566, rs12580246, rs16941724, rs16941759, rs1981517, rs2393967, rs3177647, rs3741998, rs3752630, rs441, rs4646777, rs4646778, rs4648328, rs6489824, rs7294623,	A genome-wide meta-analysis identifies 22 loci associated with eight hematological parameters in the HaemGen consortium., A meta-analysis and genome-wide association study of platelet count and mean platelet volume in	rs2393967, rs6490294

			rs7295294, rs7297760, rs7310545, rs7313773, rs7314870, rs7316563, rs7974772, rs7976557	african americans.	
Melanoma	2	CTSK, DEF8	kgp10983143, kgp10994940, kgp12565486, kgp1609468, kgp2343544, kgp2518335, kgp2973717, kgp3924204, kgp435055, kgp4786156, kgp5014129, kgp8543530, kgp9969711, rs11076649, rs11204695, rs11204725, rs11204735, rs11204737, rs12125650, rs12568757, rs4238833, rs4442808, rs4785755, rs4970926, rs6694531, rs8049897, rs8051733, rs8063761	Genome-wide association study identifies a new melanoma susceptibility locus at 1q21.3., Genome-wide association study identifies three loci associated with melanoma risk.	rs4785763, rs7412746
Menarche (age at onset)	2	ARNTL, RBM6	kgp10720473, kgp7608332, rs10766066, rs10832006, rs11022742, rs11022755, rs11605518, rs12290622, rs1481880, rs1481891, rs2279284, rs2279285, rs2279286, rs2279287, rs2681780, rs4757138, rs7107287, rs900145, rs998089	Thirty new loci for age at menarche identified by a meta-analysis of genome-wide association studies.	rs6762477, rs900145
Menopause (age at onset)	1	LY6G5C	rs1046089	A genome-wide association study of early menopause and the combined impact of identified variants., Meta-analyses identify 13 loci associated	rs1046089

				with age at menopause and highlight DNA repair and immune pathways.	
Metabolic traits	1	TRAF3IP2	kgp11051943, kgp11508579, kgp12018996, kgp12280437, kgp2633262, kgp3417858, kgp3486066, kgp3888193, kgp7861063, kgp9233618, rs10872069, rs11153290, rs11153291, rs12211763, rs240993, rs3851225, rs455650, rs455726, rs455732, rs456569, rs459147, rs459809, rs461646, rs462432, rs462779, rs4947122, rs6920798, rs6937734, rs7451051, rs7751272, rs7766610, rs7776346, rs9398272, rs9400481	Human metabolic individualit y in biomedical and pharmaceuti cal research.	rs7760535
Metabolite levels	2	CLTCL1, TRAF3IP2	kgp11051943, kgp11508579, kgp12018996, kgp12280437, kgp2633262, kgp3417858, kgp3486066, kgp3888193, kgp7851028, kgp7861063, kgp9233618, rs1018764, rs10872069, rs11153290, rs11153291, rs12211763, rs240993, rs3851225, rs455650, rs455726, rs455732, rs456569, rs459147, rs459809, rs461646, rs462432, rs462779, rs4947122, rs6920798, rs6937734, rs7451051, rs756651, rs762523, rs7751272, rs7766610, rs7776346, rs9398272, rs9400481	Genome-wide association study identifies multiple loci influencing human serum metabolite levels.	rs6900341, rs807669
Multiple sclerosis	6	CPT1B, ECGF1, HLA-DRB5,	kgp11538372, kgp24709736, rs12148, rs140522, rs140523,	Evidence for VAV2 and	rs12368653, rs140522, rs2523393,

		HLA-F, TYMP, XRCC6BP1	rs2523393, rs2782, rs4760332, rs870392, rs9271366	ZNF433 as susceptibility genes for multiple sclerosis., Genetic risk and a primary role for cell-mediated immune mechanisms in multiple sclerosis., Genome-wide association study identifies new multiple sclerosis susceptibility loci on chromosomes 12 and 20., Meta-analysis of genome scans and replication identify CD6, IRF8 and TNFRSF1A as new multiple sclerosis susceptibility loci.	rs703842, rs9271366
Multiple sclerosis (OCB status)	1	HLA-DRB5	kgp10086042, kgp10716828, kgp10791374, kgp10993269, kgp11057669, kgp11097589, kgp11111197, kgp112624, kgp11434010, kgp11488527, kgp11503156,	Oligoclonal band status in Scandinavian multiple sclerosis patients is associated with specific	rs9271640

			kgp11525948, kgp11670806, kgp11899131, kgp11951896, kgp12220543, kgp12292865, kgp12336002, kgp1497861, kgp1727184, kgp1770756, kgp1830625, kgp1840917, kgp1900289, kgp2124132, kgp2421267, kgp2618142, kgp2915226, kgp2944558, kgp3013361, kgp3097457, kgp3302220, kgp3555861, kgp3641442, kgp369526, kgp3844509, kgp398454, kgp4186203, kgp4330952, kgp4550051, kgp4630866, kgp5219378, kgp5417994, kgp5422503, kgp5522547, kgp5658501, kgp5661398, kgp5699920, kgp5786063, kgp5870137, kgp605392, kgp6621603, kgp6754068, kgp6778497, kgp6977253, kgp7045337, kgp7353290, kgp8041485, kgp8373990, kgp8432767, kgp8438749, kgp8765630, kgp8855468, kgp926303, kgp9265745, kgp9455964, kgp95572, kgp958461, kgp967253, kgp9744894, kgp9763090, kgp9797931, kgp981634, kgp9983308, kgp9990055, rs9270856, rs9271055, rs9271100, rs9271170, rs9271525	genetic risk alleles.	
Nasopharyngeal carcinoma	1	HCG4	rs2517713, rs2860580	A genome-wide association study of nasopharyngeal carcinoma identifies three new susceptibility loci., Genome-	rs2517713, rs2860580

				wide association study reveals multiple nasopharyngeal carcinoma-associated loci within the HLA region at chromosome 6p21.3.	
Natriuretic peptide levels	1	MTHFR	kgp10243024, kgp11407620, kgp1853481, kgp3278055, kgp4084024, kgp6327295, kgp8353526, rs12121543, rs12404124, rs1476413, rs1801131, rs198393, rs4846052, rs4846054, rs535107, rs6540999, rs6541003, rs7538516	Genome-wide association analysis and fine mapping of NT-proBNP level provide novel insight into the role of the MTHFR-CLCN6-NPPA-NPPB gene cluster.	rs1023252
Orofacial clefts	1	TRAF3IP3	kgp3915678, kgp4832229, kgp978599, rs126280, rs642961, rs654470, rs661849, rs680331, rs861020	Genome-wide meta-analyses of nonsyndromic cleft lip with or without cleft palate identify six new risk loci.	rs861020
Other erythrocyte phenotypes	1	EPHB4	kgp427456, kgp4978804, kgp6433169, kgp9348077, kgp9487396, kgp9573292, rs10247980, rs2293767, rs6971700	Multiple loci influence erythrocyte phenotypes in the CHARGE	rs2075671

				Consortium.	
Pain	1	ZNF493	kgp10318834, kgp1772822, kgp2226426, kgp4391716, kgp6893948, kgp7258290, kgp7328247, kgp8819005, kgp9723099, rs11085447, rs2562456, rs2562487, rs2937174, rs540714, rs627522, rs661453, rs7247411, rs7252254, rs7252261, rs7257857, rs8103325, rs8112775	Genome-wide association study of acute post-surgical pain in humans.	rs2562456
Parkinson's disease	1	BST1	kgp11711646, rs3213710, rs4541502, rs4698119, rs4698412	Genome-wide association study identifies common variants at four loci as genetic risk factors for Parkinson's disease., Imputation of sequence variants for identification of genetic risks for Parkinson's disease: a meta-analysis of genome-wide association studies.	rs11724635, rs4538475
Plasminogen activator inhibitor type 1 levels (PAI-1)	1	EPHB4	kgp6769663, rs13241786	(Huang et al., 2012)	rs6976053
Platelet counts	6	AKAP10, KIAA2013, MFN2, NRBF2, PIK3C2A, PLOD1	kgp10931301, kgp11066860, kgp1877178, kgp2933757, kgp3743467, kgp3949208, kgp5113327, kgp6834076, kgp6880103, kgp7196136, kgp860759, kgp9741946, rs1040669,	(Gieger et al., 2011)	rs10761731, rs13300663, rs2336384, rs397969

			rs10761742, rs10864546, rs1330, rs1474868, rs1810563, rs2103876, rs2108978, rs214105, rs2273291, rs3753579, rs3818157, rs3820189, rs4240897, rs4845892, rs7098614, rs730123, rs7550536, rs7551874, rs7923609		
Primary biliary cirrhosis	1	MANBA	kgp10218786, kgp10832301, kgp11307691, kgp11889234, kgp12443279, kgp4264636, kgp5801843, kgp5963878, kgp611096, kgp6261087, kgp7889616, kgp857540, kgp8863073, rs1054029, rs1054037, rs2272697, rs228611, rs228614, rs228618, rs2866413, rs2866414, rs4013, rs7665090	Genome-wide association study identifies 12 new susceptibility loci for primary biliary cirrhosis.	rs7665090
Proinsulin levels	2	MYBPC3, SPI1	kgp12136969, kgp210889, kgp3401301, kgp8645699, kgp9102914, kgp9959949, rs10501321, rs1051006, rs1052373, rs10769253, rs10838687, rs10838692, rs1449627, rs2290146, rs2290148, rs2305280, rs326214, rs3816725, rs3847502, rs4752979, rs4752983	Genome-wide association identifies nine common variants associated with fasting proinsulin levels and provides new insights into the pathophysiology of type 2 diabetes.	rs10838687
Prostate cancer	6	AGAP6, LILRA3, LILRB2, LOC100133875, PSORS1C3, RNF181	kgp10541711, kgp10578928, kgp10633694, kgp3337256, kgp3364293, kgp4973636, kgp5548545, kgp5757934, kgp6417769, kgp6543448, kgp6562647, kgp6770160, kgp8332030, kgp9538309,	Genome-wide association study identifies five new susceptibility loci for	rs10187424, rs103294, rs10993994, rs130067, rs3123078

			kgp9831195, rs1009, rs10187424, rs10198569, rs10206961, rs1058588, rs10993994, rs1124836, rs14242, rs1446669, rs1562322, rs17026396, rs2028900, rs2043675, rs2232745, rs2232750, rs3123078, rs3731828, rs4631830, rs6733550, rs6739015, rs6743051, rs6757263, rs699664	prostate cancer in the Japanese population., Genome-wide association study in Chinese men identifies two new prostate cancer risk loci at 9q31.2 and 19q13.4., Identification of seven new prostate cancer susceptibility loci through a genome-wide association study., Multiple loci identified in a genome-wide association study of prostate cancer., Multiple newly identified loci associated with prostate cancer susceptibility., Seven prostate	

				cancer susceptibility loci identified by a multi-stage genome-wide association study.	
Prostate-specific antigen levels	1	AGAP6	kgp10578928, kgp6562647, rs10993994, rs3123078, rs4631830	Genetic correction of PSA values using sequence variants associated with PSA levels., Genome-wide association study identified novel genetic variant on SLC45A3 gene associated with serum levels prostate-specific antigen (PSA) in a Chinese population.	rs10993994
Psoriasis	3	CAST, PPP2R3C, TRAF3IP2	kgp10902990, kgp11051943, kgp11508579, kgp11996373, kgp12018996, kgp12038012, kgp12280437, kgp12472474, kgp2156971, kgp2633262, kgp3417858, kgp3486066, kgp3888193, kgp46926, kgp4777039, kgp5549683, kgp7861063,	(Strange et al., 2010; Stuart, Nair, Ellinghaus, & Ding, 2010)	rs12586317, rs240993, rs27524

			kgp9233618, rs10142667, rs1028448, rs10872069, rs11153290, rs11153291, rs12211763, rs12590678, rs17103042, rs2254990, rs2273154, rs2295104, rs240993, rs26507, rs27037, rs27038, rs27433, rs28081, rs3809448, rs3851225, rs455650, rs455726, rs455732, rs456569, rs459147, rs459809, rs461646, rs462432, rs462779, rs4947122, rs4982254, rs6920798, rs6937734, rs7451051, rs7751272, rs7766610, rs7776346, rs9398272, rs9400481		
QT interval	1	CNOT1	kgp10241706, kgp10936141, kgp11271468, kgp1154945, kgp11711691, kgp1434796, kgp2030688, kgp2779117, kgp5112364, P1_M_061510_16_91_P, rs11866002, rs1549607, rs2090727, rs2243463, rs2280397, rs246258, rs27097, rs28307, rs37036, rs37054, rs3809594, rs3809596, rs6499962, rs7189568, rs7205071, rs8049393, rs8054895, rs863433, rs950843, rs9926577	Common variants at ten loci modulate the QT interval duration in the QTSCD Study.	rs7188697
Red blood cell traits	3	CPT1B, ECGF1, TYMP	kgp24709736, rs12148, rs140522, rs140523, rs2782	Seventy-five genetic loci influencing the human red blood cell.	rs140522
Renal function-related traits (BUN)	1	THBS3	kgp10676451, kgp4656286, kgp4859340, kgp8652154, rs11264337, rs11264338, rs11264339, rs2049805, rs2075571, rs2990245, rs3814316, rs4072037, rs4971100, rs914615	(Okada et al., 2012)	rs2049805

Rheumatoid arthritis	6	ARAP1, CENTD2, HLA-F, LOC253039, LY6G5C, RNASET2	GA028274, kgp11075358, kgp11138329, kgp12482760, kgp1753996, kgp2850730, kgp3245966, kgp464529, kgp4862811, kgp668171, kgp766516, kgp8167293, kgp818352, kgp8303535, kgp9111185, kgp9568439, rs1014530, rs10484530, rs10818488, rs10946204, rs12183084, rs12529876, rs12576058, rs1610677, rs1872126, rs2001114, rs204295, rs2282860, rs2301436, rs239935, rs341053, rs3761846, rs3761847, rs6902119, rs6907666, rs6921588, rs7021049, rs7749278, rs805297, rs900613, rs917770, rs933243, rs9355610, rs9459836	Common variants at the promoter region of the APOM confer a risk of rheumatoid arthritis., Genome-wide association study meta-analysis identifies seven new rheumatoid arthritis risk loci., Meta-analysis identifies nine new loci associated with rheumatoid arthritis in the Japanese population., Pathway-driven gene stability selection of two rheumatoid arthritis GWAS identifies and validates new susceptibility genes in receptor mediated signalling pathways., REL,	rs1610677, rs3093023, rs3761847, rs3781913, rs805297, rs881375

				encoding a member of the NF-kappaB family of transcription factors, is a newly defined risk locus for rheumatoid arthritis., TRAF1-C5 as a risk locus for rheumatoid arthritis--a genomewide study.	
Sarcoidosis	1	TRPT1	rs510372	(Fischer et al., 2012)	rs479777
Schizophrenia	1	LSM1	kgp1042537, kgp11341169, kgp1175064, kgp1807986, kgp1938999, kgp240436, kgp3569684, kgp3766418, kgp4480356, kgp5602620, kgp6558176, kgp6609897, kgp6925651, kgp7981588, kgp8978894, rs10095753, rs10101168, rs10107763, rs10958670, rs11774214, rs11783967, rs11986274, rs12678205, rs13317, rs1488934, rs1488935, rs1488936, rs1599918, rs16887244, rs16887273, rs17435276, rs1906672, rs2234555, rs2280847, rs2306899, rs28439472, rs2898674, rs4379415, rs4537271, rs7821392, rs7837189, rs7841617, rs7845911	Common variants on 8p12 and 1q24.2 confer risk of schizophrenia.	rs16887244
Sphingolipid levels	1	LOC255167	rs1566039	(Demirkan et al., 2012)	rs1566039
Stevens-Johnson syndrome and toxic epidermal	1	HCG27	rs3130501	(Génin et al., 2011)	rs3130501

necrolysis (SJS-TEN)					
Systemic lupus erythematosus	2	HLA-DRB5, UBE2L3	kgp10086042, kgp10791374, kgp10993269, kgp11057669, kgp11097589, kgp11111197, kgp112624, kgp11434010, kgp11488527, kgp11503156, kgp11525948, kgp11670806, kgp11899131, kgp11951896, kgp12220543, kgp12264864, kgp12292865, kgp12336002, kgp1497861, kgp1727184, kgp1770756, kgp1830625, kgp1900289, kgp2124132, kgp2421267, kgp2618142, kgp2915226, kgp2944558, kgp296173, kgp3013361, kgp3073572, kgp3097457, kgp3178602, kgp3302220, kgp334230, kgp3555861, kgp3641442, kgp369526, kgp3844509, kgp398454, kgp3989844, kgp399362, kgp4135828, kgp414481, kgp4186203, kgp4275121, kgp4330952, kgp4483134, kgp4550051, kgp4610830, kgp4630866, kgp5203504, kgp5219378, kgp5327376, kgp5417994, kgp5422503, kgp5522547, kgp5658501, kgp5661398, kgp5699920, kgp5786063, kgp5870137, kgp594723, kgp605392, kgp6621603, kgp6754068, kgp6778497, kgp6977253, kgp7045337, kgp732004, kgp7353290, kgp7612853, kgp8041485, kgp8064780, kgp8173522, kgp8373990, kgp8432767, kgp8438749, kgp8765630, kgp8855468, kgp926303,	Genome-wide association study in a Chinese Han population identifies nine new susceptibility loci for systemic lupus erythematosus., Meta-analysis followed by replication identifies loci in or near CDKN1B, TET3, CD80, DRAM1, and ARID5B as associated with systemic lupus erythematosus in Asians.	rs131654, rs9270984, rs9271100

			kgp9265745, kgp9455964, kgp95572, kgp958461, kgp967253, kgp9744894, kgp9751502, kgp9763090, kgp9797931, kgp981634, kgp98585, kgp9983308, kgp9990055, rs131654, rs9270856, rs9270984, rs9271055, rs9271100, rs9271170, rs9271525		
Systolic blood pressure	5	C15orf17, CSK, LY6G5C, MTHFR, USMG5	kgp10151543, kgp12435695, kgp1759804, kgp2626044, kgp3256932, kgp3959331, kgp4017153, kgp452259, kgp5856185, kgp7193421, kgp7228049, kgp7588133, kgp9173836, rs11191593, rs11636952, rs12567136, rs13306553, rs13306556, rs13306561, rs1378940, rs1378942, rs1537516, rs17037390, rs17037396, rs17037397, rs17037425, rs17367504, rs17421560, rs2050265, rs2066470, rs2076003, rs2076004, rs2168518, rs2236797, rs2272803, rs2274976, rs3737967, rs3753584, rs7537765, rs7553194, rs805303	(Ehret et al., 2011; Levy et al., 2009; Newton-Cheh et al., 2009)	rs1004467, rs1378942, rs17367504, rs805303
Triglycerides	1	NRBF2	kgp10931301, kgp1877178, rs10761742, rs7098614, rs7923609	(Teslovich et al., 2010)	rs10761731
Tumor biomarkers	1	FAM3B	kgp1207290, kgp8349177, rs441810	A genome wide association study of genetic loci that influence tumour biomarkers cancer antigen 19-9, carcinoembryonic antigen and α	rs441810

				fetoprotein and their associations with cancer risk.	
Type 1 diabetes	5	HLA-DRB5, HOXA5, LOC650557, SULT1A2, TUFM	GA023702, GA023720, GA031269, kgp10004293, kgp10068472, kgp1028867, kgp10300738, kgp10438878, kgp11183228, kgp11545931, kgp11579019, kgp12156514, kgp1576216, kgp17263711, kgp1774491, kgp2161314, kgp2275154, kgp2281699, kgp2647799, kgp300464, kgp3193328, kgp3705618, kgp3855964, kgp391239, kgp4151029, kgp4501731, kgp4618664, kgp5024284, kgp519135, kgp528534, kgp5334779, kgp6110830, kgp648083, kgp652537, kgp7104286, kgp7825153, kgp7927966, kgp8004961, kgp8048732, kgp8174959, kgp8234243, kgp8381958, kgp8392648, kgp9414959, kgp9558281, kgp9793551, rs1063355, rs2253415, rs2264803, rs2462904, rs2522824, rs2648076, rs4788069, rs9273349	(Barrett et al., 2009; Cooper et al., 2008; Wellcome, Case, & Consortium, 2007)	rs4788084, rs7804356, rs9272346
Type 1 diabetes autoantibodies	2	SULT1A2, TUFM	GA031269, rs4788069	(Plagnol et al., 2011)	rs4788084
Type 2 diabetes	2	MAEA, PSTPIP1	kgp1865546, kgp2953632, kgp5092636, kgp5865861, kgp6242706, kgp8347386, kgp9009344, kgp9486298, kgp9924176, kgp997727, rs11247980, rs11632941, rs11933855, rs17164620, rs3743478, rs3806754, rs4243045, rs6599300	(Cho et al., 2012; Kooner et al., 2011; Perry et al., 2012)	rs6815464, rs7178572
Ulcerative colitis	6	HLA-DRB5, LOC650557,	GA018284, kgp10720615, kgp12077599, kgp2108660,	Genome-wide	rs35675666, rs3774959,

		MANBA, NFKB1, PIM3, UTS2	kgp22797454, kgp7023928, kgp7525711, kgp789930, kgp9442604, rs1020759, rs11733293, rs118882, rs12758337, rs137862, rs1585213, rs1598859, rs1599961, rs1609798, rs161802, rs17032705, rs17367289, rs225100, rs225106, rs225119, rs225132, rs226242, rs226251, rs230491, rs230498, rs230515, rs230519, rs230528, rs230535, rs230541, rs230542, rs2493215, rs2903282, rs397349, rs400736, rs442862, rs4648011, rs4698863, rs747559, rs9271366, rs980455	association study for ulcerative colitis identifies risk loci at 7q22 and 22q13 (IL17REL)., Genome-wide association study of ulcerative colitis in Koreans suggests extensive overlapping of genetic susceptibility with Caucasians., Host-microbe interactions have shaped the genetic architecture of inflammatory bowel disease., Meta-analysis identifies 29 additional ulcerative colitis risk loci, increasing the number of confirmed associations to 47.	rs5771069, rs6927022, rs9271366
Urate levels	2	SF1, THBS3	kgp10265459, kgp10676451, kgp12119258, kgp2279666, kgp3983980, kgp4656286,	(Köttgen et al., 2013; Tin et al., 2011)	rs11264341, rs606458

			kgp4689319, kgp4859340, kgp498158, kgp5453329, kgp7644840, kgp8652154, kgp8756104, rs10897525, rs11264337, rs11264338, rs11264339, rs1143937, rs17146121, rs2049805, rs2075571, rs2990245, rs3741398, rs3809076, rs3814316, rs4072037, rs474707, rs477549, rs483962, rs489192, rs4971100, rs523200, rs532747, rs606458, rs624975, rs625172, rs637332, rs677298, rs7939965, rs914615		
Ventricular conduction	1	UBE2L3	rs131654	(Sotoodehnia et al., 2010)	rs13165478
Vitiligo	2	HCG4, RNASET2	rs2236313, rs6904029	Genome-wide association study for vitiligo identifies susceptibility loci at 6q27 and the MHC., Variant of TYR and autoimmunity susceptibility loci in generalized vitiligo.	rs2236313, rs6904029
Weight	1	TUFM	kgp5388595, rs8049439	(Thorleifsson et al., 2009)	rs7498665

Supplementary Table 2: Gene expression comparison between involved skin from cases and normal skin from controls for the 43 genes reported by IPA as being associated with psoriasis (GEO dataset GSE133555). Data used to generate Supplementary figure 1.

ID	Symbol	logFC	AveExpr	t	B	P.Value	FDR
201012_at	ANXA1	0.21	-0.01	4.57	2.20	8.84E-06	1.25E-05
233011_at	ANXA1	-0.08	-0.01	-1.16	-7.07	2.49E-01	2.72E-01
209906_at	C3AR1	0.35	-0.01	5.17	4.76	6.26E-07	9.21E-07
200696_s_at	GSN	-0.69	0.02	-15.82	70.89	6.73E-36	3.26E-35
214040_s_at	GSN	-0.72	0.02	-7.93	19.31	2.26E-13	4.55E-13
227957_at	GSN	-0.02	0.00	-0.42	-7.65	6.77E-01	6.93E-01
227958_s_at	GSN	0.01	0.00	0.35	-7.67	7.24E-01	7.36E-01
209827_s_at	IL16	-0.32	0.00	-6.52	11.41	6.91E-10	1.19E-09
209828_s_at	IL16	-0.30	0.00	-6.08	9.15	6.87E-09	1.10E-08
1555016_at	IL16	0.03	0.00	0.57	-7.57	5.71E-01	5.98E-01
213287_s_at	KRT10	-0.07	0.01	-3.48	-1.84	6.21E-04	8.13E-04
210633_x_at	KRT10	-0.03	0.01	-1.62	-6.43	1.07E-01	1.22E-01
207023_x_at	KRT10	-0.01	0.01	-0.54	-7.59	5.91E-01	6.14E-01
206851_at	RNASE3	-0.04	0.00	-1.18	-7.04	2.39E-01	2.63E-01
218921_at	SIGIRR	-0.04	0.00	-0.96	-7.27	3.37E-01	3.62E-01
237252_at	THBD	1.10	-0.01	12.68	49.89	9.55E-27	3.38E-26
203888_at	THBD	0.62	0.00	9.97	31.97	6.38E-19	1.64E-18
203887_s_at	THBD	0.58	0.00	9.42	28.48	2.15E-17	5.20E-17
208760_at	UBE2I	-0.83	0.01	-17.87	84.32	9.62E-42	6.30E-41
213535_s_at	UBE2I	0.24	0.00	8.78	24.45	1.25E-15	2.77E-15
1558088_a_at	UBE2I	-0.16	0.00	-6.38	10.68	1.45E-09	2.43E-09
211008_s_at	UBE2I	-0.05	0.00	-1.26	-6.94	2.09E-01	2.34E-01
233360_at	UBE2I	-0.05	0.00	-1.05	-7.18	2.94E-01	3.19E-01
204393_s_at	ACPP	1.77	-0.01	16.94	78.34	3.87E-39	2.20E-38
237030_at	ACPP	0.66	0.00	9.72	30.34	3.29E-18	8.29E-18
200974_at	ACTA2	-0.66	0.00	-9.98	32.07	5.79E-19	1.52E-18
243140_at	ACTA2	-0.91	0.01	-8.46	22.52	8.84E-15	1.90E-14
215787_at	ACTA2	0.02	0.00	0.78	-7.43	4.35E-01	4.63E-01
210873_x_at	APOBEC3A	1.30	-0.01	10.45	35.06	2.85E-20	8.11E-20
206177_s_at	ARG1	1.87	-0.02	17.05	79.01	1.98E-39	1.23E-38
231662_at	ARG1	0.63	-0.01	12.44	48.27	4.84E-26	1.67E-25
231663_s_at	ARG1	0.46	-0.01	11.21	40.08	1.83E-22	5.84E-22
231665_at	ARG1	0.09	0.00	6.27	10.09	2.66E-09	4.40E-09
205416_s_at	ATXN3	0.54	-0.01	10.20	33.49	1.38E-19	3.77E-19
235240_at	ATXN3	-0.36	0.00	-9.08	26.33	1.87E-16	4.38E-16
205415_s_at	ATXN3	0.42	-0.01	6.81	12.97	1.41E-10	2.50E-10
233182_x_at	ATXN3	-0.17	0.00	-6.07	9.09	7.37E-09	1.16E-08
216657_at	ATXN3	-0.16	0.00	-5.65	7.03	6.04E-08	9.31E-08
238723_at	ATXN3	-0.34	0.00	-5.18	4.80	6.02E-07	8.97E-07
217321_x_at	ATXN3	-0.09	0.00	-5.14	4.62	7.24E-07	1.05E-06

1554406_a_at	CLEC7A	2.01	-0.02	28.32	145.13	2.94E-68	5.51E-67
1555756_a_at	CLEC7A	3.12	-0.03	25.81	131.82	1.92E-62	2.29E-61
1555214_a_at	CLEC7A	1.59	0.00	22.86	115.08	3.79E-55	3.82E-54
221698_s_at	CLEC7A	1.94	-0.02	21.24	105.43	6.09E-51	4.20E-50
1555213_a_at	CLEC7A	0.07	0.00	1.98	-5.79	4.93E-02	5.82E-02
241098_at	CLEC7A	0.02	0.00	1.53	-6.57	1.28E-01	1.45E-01
201201_at	CSTB	0.78	-0.01	14.31	60.86	1.58E-31	6.26E-31
236449_at	CSTB	0.26	-0.01	3.55	-1.62	4.89E-04	6.47E-04
200838_at	CTSB	0.83	-0.01	16.89	77.98	5.51E-39	3.01E-38
200839_s_at	CTSB	0.67	-0.01	16.59	76.01	3.99E-38	2.01E-37
227961_at	CTSB	0.48	0.00	9.43	28.53	2.04E-17	5.05E-17
213274_s_at	CTSB	0.54	-0.01	9.01	25.86	3.02E-16	6.82E-16
213275_x_at	CTSB	0.24	-0.01	5.53	6.44	1.11E-07	1.69E-07
202450_s_at	CTSK	-0.31	0.01	-4.52	1.96	1.14E-05	1.57E-05
202902_s_at	CTSS	0.65	-0.02	10.06	32.54	3.60E-19	9.62E-19
202901_x_at	CTSS	0.53	-0.02	6.81	12.98	1.39E-10	2.49E-10
232617_at	CTSS	0.37	-0.01	6.21	9.78	3.61E-09	5.92E-09
202806_at	DBN1	-1.02	0.01	-14.47	61.95	5.30E-32	2.17E-31
217025_s_at	DBN1	-0.78	0.01	-11.88	44.53	2.09E-24	6.85E-24
208708_x_at	EIF5	1.08	-0.01	23.38	118.13	1.79E-56	1.96E-55
208290_s_at	EIF5	1.17	-0.01	21.62	107.73	6.09E-52	4.69E-51
208706_s_at	EIF5	0.31	0.00	9.05	26.13	2.30E-16	5.29E-16
208705_s_at	EIF5	0.48	0.00	7.67	17.81	1.03E-12	1.99E-12
208707_at	EIF5	0.20	0.00	4.51	1.93	1.18E-05	1.61E-05
213757_at	EIF5A	0.53	0.00	5.74	7.43	4.01E-08	6.26E-08
201123_s_at	EIF5A	0.65	-0.01	4.19	0.66	4.41E-05	5.89E-05
213753_x_at	EIF5A	0.18	-0.01	1.92	-5.90	5.62E-02	6.58E-02
201122_x_at	EIF5A	0.14	-0.01	1.24	-6.97	2.18E-01	2.42E-01
229390_at	FAM26F	1.36	-0.03	13.93	58.28	2.10E-30	7.73E-30
229391_s_at	FAM26F	1.21	-0.03	13.93	58.27	2.12E-30	7.73E-30
228362_s_at	FAM26F	0.16	0.00	7.16	14.92	1.94E-11	3.58E-11
229915_at	FAM26F	0.10	0.00	3.34	-2.31	1.02E-03	1.30E-03
35820_at	GM2A	1.86	-0.02	27.11	138.80	1.72E-65	2.82E-64
212737_at	GM2A	1.68	-0.02	25.96	132.62	8.52E-63	1.24E-61
235678_at	GM2A	1.67	-0.02	22.64	113.81	1.36E-54	1.27E-53
209727_at	GM2A	1.91	-0.02	22.53	113.16	2.62E-54	2.29E-53
33646_g_at	GM2A	2.23	-0.02	22.35	112.07	7.80E-54	6.39E-53
215891_s_at	GM2A	2.33	-0.02	21.36	106.18	2.89E-51	2.11E-50
215890_at	GM2A	0.29	-0.01	8.66	23.72	2.62E-15	5.73E-15
205349_at	GNA15	1.39	-0.01	25.93	132.45	1.02E-62	1.33E-61
206643_at	HAL	2.24	-0.02	17.02	78.85	2.31E-39	1.38E-38
237967_at	HAL	0.11	0.00	6.46	11.09	9.53E-10	1.62E-09
210514_x_at	HLA-G	0.35	0.00	11.13	39.56	3.06E-22	9.55E-22
211528_x_at	HLA-G	0.28	0.00	9.12	26.55	1.50E-16	3.58E-16
211529_x_at	HLA-G	0.25	0.00	7.15	14.82	2.15E-11	3.91E-11
211530_x_at	HLA-G	0.17	0.00	4.55	2.08	1.00E-05	1.39E-05
203665_at	HMOX1	1.12	-0.02	15.33	67.65	1.74E-34	7.58E-34
200699_at	KDELR2	-0.33	0.00	-7.75	18.29	6.32E-13	1.24E-12

200700_s_at	KDELR2	0.35	0.00	7.59	17.34	1.67E-12	3.16E-12
200698_at	KDELR2	-0.07	0.00	-1.90	-5.94	5.92E-02	6.86E-02
217388_s_at	KYNU	4.67	-0.04	40.90	201.85	4.00E-93	2.62E-91
210663_s_at	KYNU	3.33	-0.03	38.17	190.84	2.80E-88	1.22E-86
204385_at	KYNU	2.55	-0.03	33.19	168.99	1.07E-78	3.52E-77
210662_at	KYNU	0.27	0.00	10.75	37.06	3.82E-21	1.16E-20
241305_at	KYNU	0.05	0.00	2.93	-3.54	3.88E-03	4.79E-03
229937_x_at	LILRB1	0.52	-0.04	10.49	35.32	2.19E-20	6.37E-20
207104_x_at	LILRB1	0.37	-0.01	7.48	16.74	3.07E-12	5.74E-12
211336_x_at	LILRB1	0.33	-0.01	6.59	11.79	4.69E-10	8.18E-10
209072_at	MBP	-0.49	0.01	-8.24	21.19	3.37E-14	7.13E-14
225407_at	MBP	-0.19	0.00	-5.32	5.44	3.09E-07	4.65E-07
228938_at	MBP	0.12	0.00	4.39	1.44	1.96E-05	2.65E-05
210136_at	MBP	-0.25	-0.01	-3.14	-2.91	1.97E-03	2.48E-03
1554544_a_at	MBP	-0.16	-0.01	-2.13	-5.48	3.42E-02	4.11E-02
225408_at	MBP	-0.08	0.01	-1.75	-6.20	8.11E-02	9.32E-02
207323_s_at	MBP	-0.02	0.00	-0.46	-7.63	6.43E-01	6.64E-01
207238_s_at	PTPRC	0.89	-0.02	10.55	35.73	1.44E-20	4.30E-20
212588_at	PTPRC	0.99	-0.02	10.21	33.51	1.35E-19	3.76E-19
212587_s_at	PTPRC	0.79	-0.03	8.00	19.73	1.47E-13	3.01E-13
1569830_at	PTPRC	0.06	-0.01	2.84	-3.77	4.99E-03	6.11E-03
1552480_s_at	PTPRC	0.03	0.00	2.13	-5.49	3.46E-02	4.13E-02
216834_at	RGS1	2.80	-0.04	30.79	157.55	1.10E-73	2.88E-72
202988_s_at	RGS1	1.00	-0.02	16.74	76.99	1.49E-38	7.79E-38
202989_at	RGS1	0.05	0.00	3.48	-1.87	6.38E-04	8.27E-04
205863_at	S100A12	5.83	-0.06	43.07	210.20	8.35E-97	1.09E-94
204351_at	S100P	1.26	-0.01	8.11	20.39	7.54E-14	1.57E-13
201583_s_at	SEC23B	0.54	-0.01	11.93	44.85	1.52E-24	5.09E-24
210293_s_at	SEC23B	0.34	-0.01	7.90	19.15	2.66E-13	5.29E-13
201582_at	SEC23B	0.23	-0.01	3.47	-1.89	6.56E-04	8.42E-04
206702_at	TEK	-0.29	0.01	-4.80	3.13	3.35E-06	4.77E-06
217711_at	TEK	0.00	0.00	0.09	-7.73	9.32E-01	9.32E-01
204858_s_at	TYMP	2.60	-0.03	29.98	153.59	5.90E-72	1.29E-70
217497_at	TYMP	1.49	-0.01	15.70	70.12	1.47E-35	6.88E-35
1558549_s_at	VNN1	0.86	-0.01	15.40	68.15	1.06E-34	4.77E-34
205844_at	VNN1	1.74	-0.01	14.99	65.42	1.64E-33	6.92E-33
202112_at	VWF	-0.18	0.01	-3.00	-3.33	3.08E-03	3.84E-03
233980_s_at	VWF	-0.01	0.00	-0.21	-7.71	8.30E-01	8.37E-01
208743_s_at	YWHAB	0.24	0.00	6.19	9.69	3.98E-09	6.43E-09
217718_s_at	YWHAB	-0.04	0.00	-2.44	-4.79	1.55E-02	1.88E-02
217717_s_at	YWHAB	0.02	0.00	0.60	-7.55	5.47E-01	5.78E-01
206059_at	ZNF91	-1.33	0.02	-14.11	59.49	6.28E-31	2.42E-30
236128_at	ZNF91	-0.34	0.00	-4.85	3.35	2.68E-06	3.86E-06

Supplementary Table 3. Gene expression comparison between involved skin from cases and normal skin from controls for the 43 genes reported by IPA as being associated with psoriasis (GEO dataset GSE14905). Data used to generate Supplementary figure 2.

ID	Symbol	logFC	AveExpr	t	B	P.Value	FDR
233011_at	ANXA1	-3.39	5.11	-7.77	15.51	2.24E-11	1.17E-10
200696_s_at	GSN	-1.41	11.17	-5.81	7.09	1.20E-07	4.03E-07
1558088_a_at	UBE2I	-0.30	5.33	-3.30	-1.94	1.45E-03	2.98E-03
201012_at	ANXA1	0.38	11.88	2.69	-3.57	8.58E-03	1.58E-02
203888_at	THBD	-0.38	8.74	-2.00	-5.11	4.93E-02	7.60E-02
203887_s_at	THBD	-0.33	9.35	-1.91	-5.27	5.98E-02	9.11E-02
213287_s_at	KRT10	0.09	15.22	1.88	-5.32	6.31E-02	9.50E-02
207023_x_at	KRT10	0.10	14.88	1.86	-5.36	6.61E-02	9.73E-02
213535_s_at	UBE2I	0.23	9.68	1.82	-5.44	7.29E-02	1.04E-01
210633_x_at	KRT10	0.07	14.39	1.61	-5.79	1.12E-01	1.56E-01
237252_at	THBD	0.32	6.45	1.49	-5.96	1.40E-01	1.90E-01
209906_at	C3AR1	0.38	5.58	1.45	-6.03	1.52E-01	2.03E-01
208760_at	UBE2I	-0.20	6.53	-1.24	-6.31	2.20E-01	2.85E-01
233360_at	UBE2I	-0.18	3.38	-1.21	-6.33	2.29E-01	2.93E-01
211008_s_at	UBE2I	0.01	2.25	1.19	-6.37	2.39E-01	3.02E-01
218921_at	SIGIRR	-0.13	4.54	-1.06	-6.51	2.93E-01	3.55E-01
1555016_at	IL16	-0.03	2.37	-0.80	-6.74	4.25E-01	5.02E-01
227957_at	GSN	0.05	2.37	0.58	-6.90	5.63E-01	6.36E-01
227958_s_at	GSN	-0.01	2.18	-0.41	-6.98	6.83E-01	7.59E-01
209827_s_at	IL16	0.06	5.99	0.32	-7.02	7.53E-01	8.02E-01
209828_s_at	IL16	-0.04	3.76	-0.29	-7.02	7.74E-01	8.11E-01
206851_at	RNASE3	0.00	2.08	0.16	-7.05	8.72E-01	8.92E-01
214040_s_at	GSN	-0.02	7.35	-0.06	-7.06	9.56E-01	9.70E-01
205863_at	S100A12	6.68	5.19	18.40	58.74	1.78E-30	2.33E-28
1554406_a_at	CLEC7A	3.10	5.49	16.08	50.44	8.82E-27	5.46E-25
1555756_a_at	CLEC7A	4.36	5.78	15.99	50.10	1.25E-26	5.46E-25
212737_at	GM2A	2.02	10.67	15.87	49.66	1.95E-26	6.39E-25
35820_at	GM2A	2.09	9.98	15.73	49.12	3.38E-26	8.86E-25
217388_s_at	KYNU	4.62	6.69	15.04	46.49	4.94E-25	1.08E-23
221698_s_at	CLEC7A	3.40	6.88	14.52	44.45	3.93E-24	7.35E-23
210663_s_at	KYNU	4.18	5.15	14.39	43.93	6.70E-24	1.10E-22
204385_at	KYNU	3.23	5.17	13.62	40.87	1.50E-22	2.18E-21
208708_x_at	EIF5	1.67	9.89	13.03	38.43	1.80E-21	2.36E-20
208705_s_at	EIF5	1.01	11.46	11.98	34.03	1.56E-19	1.86E-18
204393_s_at	ACPP	2.76	8.39	11.94	33.84	1.89E-19	2.06E-18
208290_s_at	EIF5	1.78	8.59	11.63	32.55	7.00E-19	7.05E-18
235678_at	GM2A	1.71	6.77	11.43	31.67	1.72E-18	1.61E-17
208706_s_at	EIF5	0.97	10.58	11.37	31.42	2.21E-18	1.93E-17
206643_at	HAL	3.56	9.97	11.18	30.59	5.12E-18	4.19E-17
205844_at	VNN1	3.03	5.02	11.08	30.15	7.98E-18	6.15E-17
206177_s_at	ARG1	3.49	11.24	9.65	23.84	4.76E-15	3.46E-14

204858_s_at	TYMP	3.05	5.14	9.60	23.65	5.76E-15	3.97E-14
208707_at	EIF5	1.07	5.60	9.54	23.36	7.76E-15	5.08E-14
201201_at	CSTB	1.34	12.89	9.28	22.24	2.42E-14	1.51E-13
208743_s_at	YWHAB	0.68	11.13	9.23	21.99	3.11E-14	1.85E-13
217497_at	TYMP	1.77	5.44	8.48	18.64	9.32E-13	5.31E-12
1558549_s_at	VNN1	1.96	3.83	8.28	17.77	2.24E-12	1.22E-11
33646_g_at	GM2A	3.26	6.38	7.71	15.22	2.98E-11	1.50E-10
229390_at	FAM26F	1.98	6.00	7.56	14.55	5.91E-11	2.87E-10
236449_at	CSTB	1.22	5.36	7.53	14.42	6.71E-11	3.14E-10
215891_s_at	GM2A	3.08	6.90	7.51	14.34	7.32E-11	3.31E-10
229391_s_at	FAM26F	1.70	5.14	7.46	14.13	9.05E-11	3.95E-10
209727_at	GM2A	2.78	7.22	7.21	13.04	2.74E-10	1.16E-09
203665_at	HMOX1	1.71	8.41	7.16	12.79	3.55E-10	1.45E-09
210873_x_at	APOBEC 3A	2.32	3.76	6.80	11.24	1.71E-09	6.79E-09
231663_s_at	ARG1	1.00	2.65	6.62	10.45	3.85E-09	1.48E-08
1555214_a_at	CLEC7A	0.90	2.94	6.23	8.79	2.09E-08	7.82E-08
243140_at	ACTA2	-1.93	4.14	-6.22	8.75	2.17E-08	7.90E-08
231662_at	ARG1	1.10	3.42	6.15	8.49	2.85E-08	1.01E-07
200839_s_at	CTSB	0.80	10.65	6.07	8.15	4.02E-08	1.39E-07
212588_at	PTPRC	1.64	7.23	5.80	7.03	1.26E-07	4.13E-07
200838_at	CTSB	0.86	10.25	5.73	6.74	1.71E-07	5.46E-07
205349_at	GNA15	0.99	8.20	5.66	6.47	2.26E-07	7.05E-07
202806_at	DBN1	-1.79	6.30	-5.51	5.85	4.24E-07	1.29E-06
206702_at	TEK	-0.96	4.18	-5.38	5.34	7.23E-07	2.15E-06
200700_s_at	KDELR2	0.64	9.29	5.29	4.96	1.06E-06	3.09E-06
206059_at	ZNF91	-0.88	9.42	-5.13	4.37	1.95E-06	5.44E-06
209072_at	MBP	-0.86	4.15	-5.13	4.37	1.95E-06	5.44E-06
202988_s_at	RGS1	-1.71	4.95	-5.06	4.09	2.62E-06	7.15E-06
207238_s_at	PTPRC	1.56	6.14	4.90	3.49	4.87E-06	1.30E-05
217025_s_at	DBN1	-1.17	5.00	-4.75	2.92	8.78E-06	2.30E-05
211336_x_at	LILRB1	0.44	3.25	4.64	2.49	1.37E-05	3.52E-05
200699_at	KDELR2	-0.51	8.94	-4.58	2.29	1.68E-05	4.23E-05
210293_s_at	SEC23B	0.41	8.82	4.54	2.14	1.98E-05	4.89E-05
227961_at	CTSB	0.62	8.74	4.21	0.99	6.52E-05	1.58E-04
232617_at	CTSS	0.85	6.91	3.95	0.10	1.65E-04	3.94E-04
201583_s_at	SEC23B	0.52	7.89	3.94	0.07	1.71E-04	3.99E-04
229937_x_at	LILRB1	0.65	3.39	3.89	-0.12	2.09E-04	4.81E-04
202901_x_at	CTSS	0.76	6.82	3.80	-0.41	2.84E-04	6.40E-04
210514_x_at	HLA-G	0.68	10.52	3.70	-0.72	3.96E-04	8.80E-04
202902_s_at	CTSS	1.00	6.79	3.61	-1.01	5.36E-04	1.17E-03
213274_s_at	CTSB	0.52	7.78	3.60	-1.02	5.45E-04	1.17E-03
216834_at	RGS1	-1.45	6.15	-3.58	-1.10	5.93E-04	1.25E-03
207104_x_at	LILRB1	0.27	2.89	3.37	-1.72	1.14E-03	2.38E-03
201123_s_at	EIF5A	1.83	8.61	3.21	-2.19	1.89E-03	3.81E-03
210662_at	KYNU	0.28	3.24	3.04	-2.68	3.23E-03	6.41E-03
200974_at	ACTA2	-0.95	11.81	-2.98	-2.83	3.81E-03	7.45E-03

231665_at	ARG1	0.14	2.70	2.92	-3.00	4.57E-03	8.80E-03
202112_at	VWF	-0.74	8.70	-2.89	-3.07	4.91E-03	9.33E-03
211528_x_at	HLA-G	0.50	11.32	2.88	-3.10	5.11E-03	9.57E-03
237030_at	ACPP	0.52	4.58	2.69	-3.60	8.80E-03	1.60E-02
211530_x_at	HLA-G	0.31	9.81	2.68	-3.61	8.93E-03	1.60E-02
211529_x_at	HLA-G	0.43	11.08	2.55	-3.93	1.28E-02	2.26E-02
217718_s_at	YWHAB	0.16	12.91	2.50	-4.03	1.43E-02	2.50E-02
228362_s_at	FAM26F	0.17	2.37	2.46	-4.13	1.60E-02	2.76E-02
215890_at	GM2A	0.12	2.19	2.35	-4.38	2.12E-02	3.61E-02
235240_at	ATXN3	-0.20	6.89	-2.33	-4.44	2.26E-02	3.79E-02
201122_x_at	EIF5A	0.54	7.67	2.31	-4.48	2.37E-02	3.94E-02
225408_at	MBP	0.29	5.01	2.24	-4.61	2.76E-02	4.52E-02
233980_s_at	VWF	-0.05	2.06	-2.13	-4.86	3.65E-02	5.91E-02
237967_at	HAL	0.20	2.55	2.07	-4.97	4.16E-02	6.65E-02
215787_at	ACTA2	-0.07	2.71	-2.04	-5.03	4.49E-02	7.06E-02
233182_x_at	ATXN3	-0.23	5.16	-2.03	-5.04	4.52E-02	7.06E-02
241098_at	CLEC7A	0.05	2.79	1.87	-5.35	6.55E-02	9.73E-02
213753_x_at	EIF5A	0.37	7.75	1.85	-5.38	6.77E-02	9.86E-02
213275_x_at	CTSB	0.24	10.69	1.82	-5.43	7.19E-02	1.04E-01
210136_at	MBP	-0.36	8.55	-1.79	-5.49	7.73E-02	1.09E-01
216657_at	ATXN3	-0.10	3.79	-1.55	-5.88	1.26E-01	1.73E-01
236128_at	ZNF91	-0.19	5.36	-1.49	-5.97	1.41E-01	1.91E-01
212587_s_at	PTPRC	0.40	7.23	1.26	-6.28	2.11E-01	2.79E-01
202989_at	RGS1	-0.04	3.24	-1.24	-6.30	2.20E-01	2.85E-01
1555213_a_at	CLEC7A	0.08	3.14	1.21	-6.34	2.31E-01	2.93E-01
225407_at	MBP	0.12	7.89	1.16	-6.39	2.49E-01	3.10E-01
229915_at	FAM26F	0.04	2.59	1.16	-6.40	2.51E-01	3.10E-01
217321_x_at	ATXN3	-0.09	3.78	-1.09	-6.48	2.81E-01	3.44E-01
205416_s_at	ATXN3	0.13	4.97	1.00	-6.57	3.20E-01	3.84E-01
213757_at	EIF5A	0.27	10.85	0.92	-6.64	3.61E-01	4.30E-01
228938_at	MBP	0.02	1.99	0.77	-6.77	4.41E-01	5.16E-01
1569830_at	PTPRC	0.02	2.61	0.75	-6.78	4.54E-01	5.23E-01
207323_s_at	MBP	-0.06	3.00	-0.75	-6.78	4.55E-01	5.23E-01
201582_at	SEC23B	0.10	5.97	0.70	-6.82	4.87E-01	5.55E-01
217711_at	TEK	0.01	2.63	0.53	-6.93	5.99E-01	6.71E-01
238723_at	ATXN3	0.06	6.80	0.38	-6.99	7.01E-01	7.68E-01
1554544_a_at	MBP	-0.08	7.03	-0.38	-6.99	7.04E-01	7.68E-01
205415_s_at	ATXN3	-0.04	8.06	-0.37	-7.00	7.09E-01	7.68E-01
202450_s_at	CTSK	-0.10	10.11	-0.35	-7.00	7.25E-01	7.79E-01
200698_at	KDELR2	0.02	9.52	0.30	-7.02	7.65E-01	8.08E-01
1552480_s_at	PTPRC	0.01	3.05	0.23	-7.04	8.17E-01	8.49E-01
204351_at	S100P	0.07	8.28	0.18	-7.05	8.54E-01	8.81E-01
241305_at	KYNU	0.00	2.51	0.04	-7.07	9.67E-01	9.70E-01
217717_s_at	YWHAB	0.00	10.34	0.04	-7.07	9.70E-01	9.70E-01

Supplementary Table 4: Enrichment analysis for GWAS catalog containing disease/trait. For each disease or trait, the number of overlaps between the genes affected by the neutrophil eQTLs (column overlap) was compared to the number of genes known to be associated with the disease or trait in the GWAS catalog (column truth) using the hypergeometric distribution. The background for the GWAS catalog was assumed to be all human genes (28,514, UCSC known genes).

disease	symbol	Overlap	Total genes	set of neutrophil eQTLs	Truth (estimated)	P-value
Alzheimer's disease	MS4A6A	1	28514	832	60	0.305
Alzheimer's disease (late onset)	MS4A6A	1	28514	832	55	0.324
Ankylosing spondylitis	CAST	1	28514	832	22	0.345
Asthma	HLA-DRB5,IL18RAP, LOC650557	3	28514	832	43	0.094
Behcet's disease	CCR3	1	28514	832	11	0.239
Beta-2 microglubulin plasma levels	BAT5	1	28514	832	7	0.171
Blond vs. brown hair color	TPCN2	1	28514	832	5	0.130
Blood pressure	C15orf17,CSK,MTHFR	3	28514	832	64	0.170
Body mass index	MYBPC3,SPI1,TUFM	3	28514	832	97	0.227
Brain imaging	CISD1,TFAM	2	28514	832	1	0.000
Brain structure	HRK,TMEM118	2	28514	832	21	0.102
Breast cancer	ECHDC1	1	28514	832	95	0.171
C4b binding protein levels	C4BPA	1	28514	832	1	0.029
Celiac disease	RGS1	1	28514	832	58	0.313
Cholesterol, total	,CPNE1	2	28514	832	79	0.269
Chronic obstructive pulmonary disease-related biomarkers	HCG27,PSORS1C3	2	28514	832	33	0.180
Cleft lip	TRAF3IP3	1	28514	832	2	0.057

Coffee consumption	CSK	1	28514	832	6	0.151
Coronary heart disease	HCG27	1	28514	832	115	0.114
Crohn's disease	,CISD1,DNLZ,ERAP2,HLA-F,IL18RAP,RNASET2,TFAM,TRPT1,TUFM	10	28514	832	137	0.005
Dengue shock syndrome		0	28514	832	4	0.888
Diastolic blood pressure	C15orf17,CSK,LY6G5C	3	28514	832	28	0.039
Drug-induced liver injury (amoxicillin-clavulanate)	HLA-DRB5,HLA-F	2	28514	832	2	0.001
Endometriosis	,CDC42	2	28514	832	25	0.129
Eosinophil counts	IL18RAP	1	28514	832	7	0.171
Epstein-Barr virus immune response (EBNA-1)	HLA-DRB5	1	28514	832	2	0.057
Eye color traits	C17orf90	1	28514	832	3	0.083
Glaucoma (primary open-angle)	PIK3C2A	1	28514	832	12	0.253
Graves' disease	HCG4,HLA-F,RNASET2	3	28514	832	18	0.013
HDL cholesterol	CDK2AP1,KCTD10,LILRA3,LOC100133875,MYBPC3,PPP1R1B,SPI1,UBE2L3	8	28514	832	101	0.007
Height	CCBL2,CLIC4,FIG4,N4BP2L2,OCEL1,PFAAP5,PIK3C2A,RFP,SMPD2	9	28514	832	324	0.133
Hippocampal volume	HRK,TMEM118	2	28514	832	5	0.008
Homocysteine levels	MFN2	1	28514	832	17	0.309

Hypertension	LY6G5C	1	28514	832	24	0.355
Hypothyroidism	VAV3	1	28514	832	18	0.318
IgA nephropathy	CD68,HCG4	2	28514	832	8	0.020
Immunoglobulin A	HLA-DRB5	1	28514	832	12	0.253
Inflammatory bowel disease	CISD1,HLA-DRB5,TFAM,UTS2	4	28514	832	113	0.185
Iris color	HERC2	1	28514	832	1	0.029
LDL cholesterol		0	28514	832	80	0.093
Leprosy	NOD2	1	28514	832	10	0.224
Lipoprotein-associated phospholipase A2 activity and mass	MS4A6A	1	28514	832	12	0.253
Liver enzyme levels	NRBF2	1	28514	832	10	0.224
Liver enzyme levels (gamma-glutamyl transferase)	CCBL2,DDT,THBS3	3	28514	832	26	0.033
Lymphoma	TAP2	1	28514	832	5	0.130
Magnesium levels	THBS3	1	28514	832	9	0.207
Mean corpuscular hemoglobin	DNASE2	1	28514	832	22	0.345
Mean corpuscular volume	DNASE2	1	28514	832	29	0.370
Mean platelet volume	C12orf47,NRBF2	2	28514	832	43	0.229
Melanoma	CTSK,DEF8	2	28514	832	17	0.074
Menarche (age at onset)	ARNTL,RBM6	2	28514	832	60	0.271
Menopause (age at onset)	LY6G5C	1	28514	832	36	0.373
Metabolic traits	TRAF3IP2	1	28514	832	47	0.351
Metabolite levels	CLTCL1,TRAF3IP2	2	28514	832	89	0.254
Multiple sclerosis	CPT1B,ECGF1,HLA-DRB5,HLA-F,TYMP,XRCC6BP1	6	28514	832	139	0.108

Multiple sclerosis (OCB status)	HLA-DRB5	1	28514	832	8	0.190
Nasopharyngeal carcinoma	HCG4	1	28514	832	7	0.171
Natriuretic peptide levels	MTHFR	1	28514	832	3	0.083
Orofacial clefts	TRAF3IP3	1	28514	832	44	0.360
Other erythrocyte phenotypes	EPHB4	1	28514	832	3	0.083
Pain	ZNF493	1	28514	832	4	0.107
Parkinson's disease	BST1	1	28514	832	58	0.313
Plasminogen activator inhibitor type 1 levels (PAI-1)	EPHB4	1	28514	832	4	0.107
Platelet counts	AKAP10,KIAA2013,MFN2,NRBF2,PIK3C2A,PLOD1	6	28514	832	68	0.011
Primary biliary cirrhosis	MANBA	1	28514	832	30	0.371
Proinsulin levels	MYBPC3,SPI1	2	28514	832	9	0.025
Prostate cancer	AGAP6,LILRA3,LILRB2,LOC100133875,PSORS1C3,RNF181	6	28514	832	88	0.029
Prostate-specific antigen levels	AGAP6	1	28514	832	5	0.130
Psoriasis	CAST,PPP2R3C,TRAF3IP2	3	28514	832	30	0.045
QT interval	CNOT1	1	28514	832	59	0.309
Red blood cell traits	CPT1B,ECGF1,TYMP	3	28514	832	51	0.125
Renal function-related traits (BUN)	THBS3	1	28514	832	15	0.289
Rheumatoid arthritis	ARAP1,CENTD2,HLA-F,LOC253039,LY6G5C,RNASET2	6	28514	832	134	0.100

Sarcoidosis	TRPT1	1	28514	832	4	0.107
Schizophrenia	LSM1	1	28514	832	115	0.114
Sphingolipid levels	LOC255167	1	28514	832	11	0.239
Stevens-Johnson syndrome and toxic epidermal necrolysis (SJS-TEN)	HCG27	1	28514	832	6	0.151
Systemic lupus erythematosus	HLA-DRB5,UBE2L3	2	28514	832	71	0.275
Systolic blood pressure	C15orf17,CSK,LY6G5C,MTHFR,USMG5	5	28514	832	28	0.001
Triglycerides	NRBF2	1	28514	832	62	0.297
Tumor biomarkers	FAM3B	1	28514	832	7	0.171
Type 1 diabetes	HLA-DRB5,HOXA5,LOC650557,SULT1A2,TUFM	5	28514	832	72	0.041
Type 1 diabetes autoantibodies	SULT1A2,TUFM	2	28514	832	24	0.123
Type 2 diabetes	MAEA,PSTPIP1	2	28514	832	142	0.135
Ulcerative colitis	HLA-DRB5,LOC650557,MANBA,NFKB1,PIM3,UTS2	6	28514	832	92	0.034
Urate levels	SF1,THBS3	2	28514	832	56	0.265
Ventricular conduction	UBE2L3	1	28514	832	0	0.000
Vitiligo	HCG4,RNASET2	2	28514	832	34	0.185
Weight	TUFM	1	28514	832	31	0.372
